# Shadowed by scale: subtle behavioral niche partitioning in two sympatric, tropical breeding albatross species

**DOI:** 10.1186/s40462-015-0060-7

**Published:** 2015-09-21

**Authors:** Melinda G. Conners, Elliott L. Hazen, Daniel P. Costa, Scott A. Shaffer

**Affiliations:** Department of Ocean Sciences, University of California Santa Cruz, 100 Shaffer Road, Santa Cruz, CA 95060 USA; Environmental Research Division, Southwest Fisheries Science Center, NOAA Fisheries, 1352 Lighthouse Avenue, Pacific Grove, CA 93950 USA; Joint Institute for Marine and Atmospheric Research, University of Hawai’i at Manoa, 1000 Pope Road, Honolulu, HI 96822 USA; Institute of Marine Sciences, University of California, Santa Cruz, CA 95060-5730 USA; Department of Biological Sciences, San José State University, One Washington Square, San Jose, CA 95192 USA

**Keywords:** Coexistence, Niche partitioning, Foraging strategies, “Sit-and-wait” foraging, Nocturnal foraging, Intraspecific variability, Behavioral plasticity, Albatrosses, *Phoebastria*

## Abstract

**Background:**

To meet the minimum energetic requirements needed to support parents and their provisioned offspring, the timing of breeding in birds typically coincides with periods of high food abundance. Seasonality and synchrony of the reproductive cycle is especially important for marine species that breed in high latitudes with seasonal booms in ocean productivity. Laysan and black-footed albatrosses breeding in the northwestern Hawaiian Islands have a dual reliance on both seasonally productive waters of high latitudes and on nutrient-poor waters of low latitudes, because their foraging ranges contract during the short but critical brood-guard stage. Therefore, these species face an additional constraint of having to negotiate nutrient-poor waters during the most energetically-demanding stage of the breeding cycle. This constriction of foraging range likely results in a higher density of foraging competitors. Thus, our aim was to understand how Hawaiian albatross partition resources both between and within species in this highly constrained breeding stage while foraging in less productive waters and simultaneously experiencing increased competition. High-precision GPS dataloggers were deployed on black-footed (Phoebastria nigripes, n=20) and Laysan (Phoebastria immutabilis, n=18) albatrosses during the brood-guard stage of the breeding season in 2006 (n=8), 2009 (n=13), 2010 (n=16) and 2012 (n=1). We used GPS data and movement analyses to identify six different behavioral states in foraging albatrosses that we then used to characterize foraging trips across individuals and species. We examined whether variations in behavior were correlated with both intrinsic factors (sex, body size, body condition) and extrinsic factors (lunar phase, wind speed, year).

**Results:**

Behavioral partitioning was revealed both between and within species in Hawaiian albatrosses. Both species were highly active during chick-brooding trips and foraged across day and night; however, Laysan albatrosses relied on foraging at night to a greater extent than black-footed albatrosses and exhibited different foraging patterns at night. For both species, foraging along direct flight paths and foraging on the water in a “sit-and-wait” strategy were just as prevalent as foraging in a searching flight mode, indicating flexibility in foraging strategies in Hawaiian albatross. Both species strongly increased drift forage at night when the lunar phase was the darkest, suggesting Hawaiian albatross feed on diel vertically-migrating prey to some extent. Black-footed albatrosses showed greater variation in foraging behavior between individuals which suggests a higher level of intra-specific competition. This behavioral variability in black-footed albatrosses was not correlated with sex or body size, but differences in body condition suggested varying efficiencies among foraging patterns. Behavioral variability in Laysan albatrosses was correlated with sex, such that females exhibited greater flight foraging than drift foraging, had longer trip durations and flew farther maximum distances from the breeding colony, but with no difference in body condition.

**Conclusion:**

Fine-scale movement data and an analysis of multiple behavioral states identified behavioral mechanisms that facilitate coexistence within a community of albatross during a critical life-history period when energetic demands are high, resources are limited, and competition for food is greatest.

**Electronic supplementary material:**

The online version of this article (doi:10.1186/s40462-015-0060-7) contains supplementary material, which is available to authorized users.

## Background

Reproductive strategies in birds arise from complex interactions between phylogenetic and morphological constraints and environmental conditions [1–4]. Seabirds, compared to other taxa, have low annual fecundity and older minimum breeding ages, accruing reproductive output over long lifespans [5, 6]. Moreover, seabirds that exploit pelagic waters and rely on efficient flight tend to have the lowest fecundities and highest rates of adult survival [5]. These species are more likely to defer breeding if the cost of reproduction reduces adult survival beyond a critical threshold [7, 8]. To meet the minimum energetic requirements for both parents and their offspring, breeding typically coincides with periods of high food abundance [1, 9, 10]. Seasonality and synchrony of the reproductive cycle is especially important in seabirds that breed in temperate and polar regions where mesoscale features enhance ocean productivity and are predictable but occur in narrow temporal windows (e.g., seasonal coastal upwelling, ice-edge blooms) [11–14]. In contrast, seabirds breeding in tropical and subtropical regions rely on less abundant, patchier resources [14, 15], and associate with sub-mesoscale features such as eddies and filaments [16–18]. Here, productivity peaks with less magnitude than in higher latitudes, and is episodic rather than confined to a single defined season [12]. Consequently, breeding of tropical seabirds, in general, shows weaker synchrony than their temperate counterparts [19, 20], is often protracted, and can occur throughout the year [21].

Black-footed (*Phoebastria nigripes*) and Laysan (*P. immutabilis*) albatrosses that breed sympatrically and synchronously in dense colonies throughout the northwestern Hawaiian islands are atypical albatrosses in their reliance on tropical feeding grounds (sea-surface temperatures ≥ 25 °C, [22]) during the brood-guard reproductive stage [23, 24], when chicks are too small to self-thermoregulate and are continually attended by at least one parent. The majority of albatross species breed in high latitudes of the southern hemisphere and match the brood-guard stage with seasonal highs in primary production in temperate or sub-polar waters [25]. Hawaiian albatrosses exploit cooler and productive waters associated with high latitudes during the longer foraging trips of the incubation and post-guard reproductive stages when they range farther from the nest. However, they are limited to warm, tropical waters near the breeding colony while caring for small vulnerable chicks that require frequent feedings [23, 24]. The brood-guard is the most energetically demanding breeding stage in birds [26, 27], and it is common for adult albatrosses to lose body mass during this period [24, 28]. It is therefore notable that Hawaiian-breeding albatrosses can rely on suboptimal feeding grounds during this particularly constrained life history stage.

**Fig. 1:**
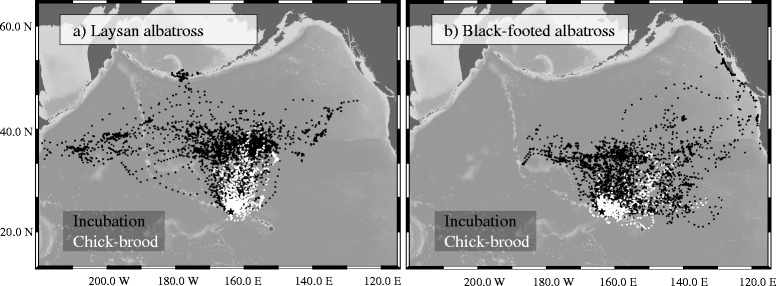
GPS and PTT locations of incubating and chick-brooding Laysan (*n* = 114, panel a) and black-footed (*n* = 118, panel b) albatross breeding at Tern Island. Tracks used for this figure were collected for this study and a concomitant long-term tracking study from 2006, 2009, 2010, 2011 and 2012. Tracks were interpolated to 1 position every 5 h. Incubating birds of both species spend the majority of time in higher latitudes in the North Pacific Transition Zone as well as coastal locations around the Pacific Rim. Clear spatial segregation between the species occurs during the incubation stage (focal range overlap of 50.8 % [24]) but spatial segregation breaks down during the brood-guard stage (focal range overlap of 75.4 % [24]). Tern Island is indicated with a star

Limited range during the brood-guard increases the potential for competitive interactions within and among species [29–31]. For most of the year, black-footed and Laysan albatrosses clearly segregate at sea with distinct habitat preferences [32–35], but during the brood-guard stage this spatial segregation breaks down and focal ranges (defined as the highest 50 % utilization distribution) of the two species overlap by 75.4 % [24] (Fig. 1). The radiation of foraging strategies among sympatrically-breeding seabirds to minimize competition and maximize resource allocation is well documented with species delineating strategies by time (e.g., [36]), space (e.g.,[37]), and morphology (e.g., [38]); however, how black-footed and Laysan albatrosses partition resources during the short but critical brood-guard stage remains unclear.

The original characterization of niche partitioning between these species — that Laysan albatrosses are nocturnal foragers of vertically-migrating squid while black-footed albatross are predominantly diurnal scavengers of fish roe and carcasses [39] — was based on two lines of evidence: 1) Laysan albatrosses have relatively high levels of rhodopsin, a light-sensitive pigment typically found in high levels among nocturnal birds (*unpublished data,* [39, 40]), and 2) Laysan albatrosses have a larger component of squid in their diet than black-footed albatrosses who have greater proportions of fish eggs and carrion [39]. But more recent diet and tracking studies do not support nocturnal foraging in Laysan albatrosses and conclude that *both* species likely have daytime biased foraging [41–43]. Additionally, activity budgets (based on data from wet-dry loggers) between the two species were found to be the most similar during the brood-guard [24], further complicating our understanding of how these albatross species partition resources in tropical waters. However, while niche separation between species can be obvious, it can also be quite subtle, like McArthur’s classic observation of congeneric warblers that forage simultaneously on insects in the same trees but at distinct heights and on different diameter branches [44]. Such subtlety can limit our understanding of niche partitioning among difficult to observe animals, such as wide-ranging pelagic marine species like albatross.

Niche partitioning within a species may also be important for central place foragers as it provides an additional mechanism for reducing competition [45–47]. Often, within species variability of foraging strategies is linked to distinct intrinsic characteristics such as sex, body size, and age [48–50], and even personality [51]; this variability is important to understand as it can affect fitness [51–54] and, therefore, population demographics. Within species niche partitioning tends to increase with the density of conspecifics, often as a result of short-term behavioral plasticity in foraging strategies [55, 56]. Consequently, quantifying the level of behavioral variability both within populations and between closely related species can illustrate the degree of competitive stressors [57].

Here, we propose, that given the reduced range during the brood-guard, behavioral niche partitioning within and between species is likely occurring at finer scales than would be observable from the resolution of geolocation or Argos data used in previous studies. We used GPS data with greater precision in spatial (<10 m error in >95 % locations) and temporal (1 fix 10 s^−1^) scales and identified six different behavioral states along albatross foraging trips to: (1) investigate behavioral partitioning between black-footed and Laysan albatrosses during the brood-guard, particularly focusing on diurnal/nocturnal differences and the influence of the lunar cycle; (2) assess and quantify the amount of within species partitioning of foraging patterns; and (3) identify intrinsic drivers (body size, sex) of those patterns and explore potential consequences (measured by body condition) of those patterns. Our ultimate objective was to understand the behavioral mechanisms facilitating coexistence within a community of albatross during a critical life-history stage when energetic demands are high, resources are limited, and competition for food is great.

## Methods

### Study species and study location

Black-footed and Laysan albatrosses are highly migratory, pelagic surface feeders thought to depend primarily upon visual and olfactory cues to find prey at the ocean surface [58]. They are the smaller-bodied of the three *Phoebastria* species breeding in the North Pacific, a trait thought to be related to the relatively lighter winds encountered in the lower latitudes of the central North Pacific during the breeding season [59]. Their populations primarily breed in the low-lying atolls of the northwestern Hawaiian Islands and, unlike albatrosses of the southern hemisphere, they initiate breeding with the winter season. Males and females are slightly sexually dimorphic, with males being marginally larger and heavier than females, but there is overlap in all of these morphometric measurements ([25], this study). Single egg clutches are laid in November and December, chicks hatch in January and February, and chicks are guarded and fed frequently by adults into March. The post-guard period, when adults extend the range and duration of foraging trips and chicks are fed infrequent meals, extends into summer until chicks fledge (July-August). The study colony at Tern Island in the French Frigate Shoals (23.870°N, 166.284°W, 712 km northwest of Kauai) supports ~4000 breeding pairs of black-footed albatrosses and ~3000 of Laysan albatrosses and is the only colony in the northwestern Hawaiian islands where the density of black-footed albatrosses is greater than that of Laysan albatrosses [60]. Although populations of both species have partially recovered since their decimation from the feather and egg trade in the early 1900s [25], they are listed as near-threatened species on the IUCN red list, and population stability for black-footed albatrosses is vulnerable to adult mortality from fisheries bycatch [61–63].This research was approved by the animal care and use committee of the University of California Santa Cruz (UCSC) and by permits from the Papahānaumokuākea Marine National Monument (PMNM-2008-006, PMNM-2009-004, PMNM-2011-015) and Special Use Permits (SUPs) from the US Fish & Wildlife Service (USFWS). 

### Tracking methods

To collect fine-scale behavioral data on foraging albatrosses during the brood-guard season, we deployed GPS dataloggers on 18 Laysan and 20 black-footed albatrosses in February and March (2006: (*n* = 8), 2009: (*n* = 13), 2010: (*n* = 16), 2012: (*n* = 1)) at Tern Island. GPS dataloggers weighed either 35 g (iGot-U GT- 120, Mobile Action Technology Inc.) or 30 g (Technosmart GiPSY-2 logger). Tags recorded positions with a temporal resolution of 1 fix every 10 s to provide fine-scale foraging behavioral data without behavioral “noise” of fine-scale flight adjustments to the wind that are recorded with smaller sampling rates [64]. GPS dataloggers were housed in unlubricated condoms and polyethylene pouches and attached to 3–5 dorsal contour feathers using TESA cloth tape. For a subset of birds of each species, 3.6 g Lotek geolocators (LAT2500) were attached to the plastic auxiliary leg band with cable ties and epoxy for concurrent research (these data were not used in this study). Total tag weight represented 1.2–1.6 % the weight of the bird, depending on species and datalogger combination; this range of percentages is well below the suggested maximum tag weight of 3 % recommended for gliding seabirds [65, 66].

### Individual characteristics

To assess intrinsic factors as potential drivers of different foraging strategies, we created a body size index for each bird from morphometrics. Lengths of culmen, tarsus, and minimum and maximum bill height were measured to ± 0.5 mm using vernier calipers*. Body Size and Body Condition:* For each species, we ran a principal components analysis (PCA) on standardized lengths of bill measurements and extracted single factor scores to construct a composite body-size index for each albatross [49]. Tarsus was not included in the body-size PCA because measurements from 2006 were consistently smaller than those from other years, likely due to measurement bias among personnel. Consequently our body size index includes only bill size, but provides an accurate score of size because bill size often correlates with body size. To increase the power of the PCA, we ran the analysis on a larger sample size of birds, from both species, by including morphometrics of birds from a concurrent study (Laysan, *n* = 163; black-footed, *n* = 167). Body mass was measured to ± 50 g on tag deployment and recovery using a spring-loaded Pesola scale. Subsequently, general body condition was calculated as an individual’s residual distance from the regression of body mass at deployment against its body size index [49]. *Sex Determination:* Sex was recorded from either 1) a visual comparison when a pair was seen together attending the nest and size differences were observable, 2) predicted from a discriminant function analysis on morphometrics [49] or 3) from DNA molecular identification. Sexes of all birds from 2006 were identified with molecular DNA, so we included tarsus lengths along with both bill measurements when calculating the discriminant function to estimate sex of birds in the other years. To increase prediction power, our discriminant function was calculated from measurements of birds of known sex (either molecular or visual confirmation) from this study as well as a concurrent study (black-footed, *n* = 43, Laysan, *n* = 35). Maximum bill depth, and culmen and tarsus lengths correctly assigned the sex of black-footed albatrosses 91 % of the time using the following regression: 7.475 maximum bill depth + 1.197 tarsus + 0.832 culmen, and 94 % of the time for Laysan albatrosses using: 2.174 culmen + 5.362 maximum bill depth + 0.697 tarsus, giving us a sample size of 12 male and 8 female black-footed and 12 male and 6 female Laysan albatrosses for subsequent analyses.

### Track analysis

All track and behavioral analyses were conducted in Matlab (2013a, The MathWorks, Inc.) with custom-built functions unless otherwise specified. High precision and accuracy (<10 m spatial error in >95 % locations) of GPS dataloggers necessitated only minimal pre-processing of tracks. A simple speed filter removed locations from raw GPS data with speeds greater than 100 km hr-1 (<0.1 % data removed); additionally, locations less than five kilometers from the breeding colony were excluded, and only a single foraging trip per bird chosen at random was included in the analysis. For a basic description of foraging trips, we calculated percent trip on water, maximum range (defined as farthest distance (km) reached from colony), trip duration (days) and total distance traveled (cumulative distance (km) between locations). To characterize movement behavior along a trip, we calculated the following parameters: flight speed (km/h), turning angle (°), drift sinuosity (0-1), and landing rate (landings/h). Birds were considered “on water” when speeds of three consecutive locations were below 6 km hr^−1^. The high sampling rate of our GPS dataloggers allowed us to identify this cutoff speed, apparent in a bimodal distribution of speeds (Additional file 1: Figure S1).

### Foraging behavior

Area-restricted search (ARS) is often used to identify foraging behavior from animal tracking data, with the logic that foraging individuals are likely to spend more time and have more sinuous paths in profitable and predictable areas [67, 68]. However, albatrosses use a suite of foraging tactics, including area-restricted search, foraging along a direct (transiting) flight path and foraging while drifting at the surface of the water using a “sit-and-wait” strategy [69–71]. To identify multiple foraging behaviors, we first calculated three behavioral metrics (residence time (ARS), landing density, and drift sinuosity) then incorporated all three metrics into a custom behavioral state classification routine outlined below.

#### *Behavioral metrics*

 1. Residence time: To identify areas of high search intensity (ARS), we calculated residence time values at every location along a track using the Pascal program (translated to Matlab) provided in Barraquand & Benhamou 2008 [72]. Residence time is a scale-dependent metric that imposes a virtual circle with a user-defined radius over each consecutive location and sums the time spent along all track segments within the circle, both forward and backward [72]. To avoid overinflating residence times in locations where birds were drifting on the water, drift segments greater than two minutes were re-discretized as if the birds were flying at a constant speed (50 km hr^−1^) (see [69]). The selection of the radius of the virtual circle in residence time analysis defines the spatial scale of identified ARS, and therefore requires justification. We selected a 10 km radius (20 km circle), because we were interested in small-scale flight searching behavior, the scale in which albatrosses would be reacting to prey through vision or scent [58], rather than large-scale reactions of birds to environmental features, as behavior at that scale has been identified previously in these species [33]. Trips were temporally re-discretized with a constant interval of 10 s. The number of steps allowed outside the virtual circle was set at 720 steps (equivalent to two hours). Finally, a Hampel filter detected and replaced local outliers along the residence time series with appropriate local values [73].

2. Landing density: Within the virtual circles (r = 10 km) calculated at each consecutive location for residence time, we additionally calculated the number of landings for all track segments that fell within the circle, both forward and backward. This approach allowed us to calculate foraging effort at the same spatial scale as flight search intensity, allowing us to decipher active versus non-active flight modes.

3. Drift sinuosity: To identify active drifts, indicating “sit-and-wait” foraging, drifting bouts were defined as locations where the bird was sitting on the water (speeds < 6 km hr^−1^) for a minimum of ten minutes (see [69]). Drifts that were separated by less than three consecutive fixes (i.e., 30 s) were combined as a single drift to avoid the artificial discretization of drifts due to short spikes of speeds above 6 km/h. We then calculated the track sinuosity of each drift as the ratio of the straight-line distance from the beginning to end of the drift and the summed distance between all consecutive locations in the drift. Sinuosity values ranged from zero, representing a completely tortuous path, to one, a perfect line. To ensure that drift sinuosity reflected albatross behavior, we tested if sinuosity was simply a reflection of wind-induced roughness of the sea-surface (wind chop), or of fine-scale looping currents induced by near-inertial oscillations. A linear mixed model regression of wind speed on drift sinuosity with species included as a fixed cofactor and individual bird as a random effect indicated no relationship between wind and drift sinuosity for either species (Additional file 2: Figure S2). Inertial currents are fine-scale looping surface currents with a strictly anti-cyclonic flow (clockwise in northern hemisphere) [74]. However, the directions of arcs in albatross drifts were both clockwise and counter-clockwise. Additionally, the loop radius of inertial currents is on average much larger (>10^3^ m, [75]) than loops in drifts of albatrosses (<10^1^ m).

#### *Behavioral state classification*

**Fig. 2:**
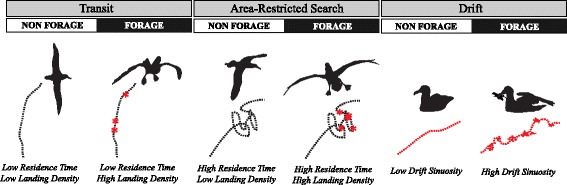
Behavioral state classification schematic showing forage and non-forage modes for two flight behaviors (Transit and ARS) and for drifting behavior. Red indicates when the bird is on water

Each location along a trip was assigned one of six behavioral states using residence time, landing density, and drift sinuosity (Fig. 2). An individual was assigned to one of two flight behaviors: ‘Transit’ or ‘ARS’, identified by either low or high residence times, respectively. A high landing density at that location indicated that the individual was in a foraging mode of that flight behavior (Fig. 2, ‘Transit Forage’ or ‘ARS Forage’), since albatross are required to land on the surface of the ocean to forage, and likely limit their landings to foraging activity given the high energetic cost of take-offs and landings [76]. Thresholds classifying residence time and landing density values as ‘High’ or ‘Low’ were unique to each individual, with ‘High’ defined as values greater than the 60^th^ percentile of an individual’s distribution of residence times and greater than the 60^th^ percentile of an individual’s distribution of landing densities. Percentile-based threshold values were selected to reflect biological and behavioral relevance based on a visual inspection of different threshold scenarios on a subset of tracks from both species. Drifts were classified as either passive drifts (low sinuosity) where the bird was resting, or active foraging drifts (high sinuosity), indicating “sit-and-wait” behavior (Fig. 2, ‘Drift Forage’) (see [36, 69]). The threshold value for drift sinuosity was fixed at 0.65 for all individuals of both species, identified as the local minima of a bimodal distribution of drift sinuosity values for both species.

### Environmental data

We classified locations as day, night, or nautical twilight using local sun zeniths extracted at each position along a foraging trip in Matlab (‘sun_position.m’). Moonrise and moonset along each trip were identified by extracting moon elevation above the horizon at each location (‘LunarAzEI.m’). Moon phase was defined as the proportion of lunar disc illuminated obtained from the U.S. Naval Observatory. Three day composites of ocean surface wind speeds derived from the quikSCAT (2006, 2009 data) and ASCAT (2010, 2012 data) scatterometer observations were extracted along tracks using the Thematic Real-time Environmental Distributed Data Services within NOAA’s Environmental Research Division.

### Statistical analyses

Except where noted, all statistical analyses were run using the ‘nlme’ [77], ‘mgcv’ [78], ‘multcomp’ [79], and’pvclust’ [80] packages in R 3.1.1 [81]. When necessary, variance structures were included in regression models to account for heterogeneity in residuals using the ‘varComb’ and ‘varIdent’ functions in the ‘nlme’ package. Significance was defined as *P* ≤ 0.05 and marginal significance as *P* ≤ 0.10. Permutational MANOVA and simper analyses [82] were run in PRIMER statistical software.

#### Movement parameters and general track characteristics

Linear mixed effect models tested differences in movement parameters (flight speeds and turning angles, landing rates, drift durations, drift sinuosities and percent of trip in flight). Species, astronomical state (day, night or twilight), and their interaction were fixed effects and individual birds were used as random factors. The fixed effects of sex and year were found insignificant and not included in final models. Landing rate and turn angle distributions were log transformed and percent trip in flight was arcsin transformed to meet assumptions of normality. Posthoc multiple comparisons using Tukey contrasts identified diurnal differences both between and within species. General track characteristics (total trip distance, maximum distance reached, mean daily distance traveled and trip duration) were compared using linear models with species, sex and year as fixed effects. Maximum distance was square root transformed before analysis.

#### Behavioral state species comparison

Multiple permutational MANOVA analyses tested for differences in behavioral composition of trip (% trip in each behavioral state) between species. Species differences were tested for both overall trip behavioral composition as well as behavioral composition of day and of night portions of trip. The resemblance matrix was calculated using Euclidean distance, an unrestricted permutation method, and Type III (partial) sums of squares. Then, a similarity percentage analysis ('simper', [82]) identified the relative contribution of behavioral states to the dissimilarity between species.

#### Effect of lunar phase on behavioral state

To understand how albatross foraging behavior responded to lunar phase, we ran a series of generalized additive mixed models. Each behavioral state was examined separately and modeled as a binomial dependent variable. Lunar phase, as an explanatory variable, was included in the model using a cyclical smoothing spline and nested under astronomical state to isolate the smoother at night, when behavior would be potentially affected by the moon. To account for the contribution of individual variability to the error term, individual bird was included as a random effect. A first order autoregressive correlation structure was incorporated to account for temporal autocorrelation.

#### Identifying and characterizing foraging patterns within species

Within each species, a hierarchical clustering analysis grouped individuals into discrete foraging patterns based on the duration of the trip (%) in each of the six behavioral states (‘pvclust’, following [83]). This method identifies significant clusters by calculating approximately unbiased (AU) p-values using multiscale bootstrap resampling. We used Euclidean distance and the Ward agglomeration method to identify significant clusters at the *P* ≥ 0.95 level, but then applied the 50 % similarity level to define population-level foraging patterns (see [83]). Once birds were clustered into overall foraging patterns, we further characterized strategies by comparing additional behavioral parameters and track characteristics between clusters. Behavioral parameters included the proportions of day and night the birds were in the foraging mode of each behavioral state (‘% Day in Transit Forage’, ‘% Night in Transit Forage’, etc.) and day and night landing rates (landings hr^−1^), while track characteristics included mean daily distance traveled (km day^−1^), total cumulative distance traveled (km), maximum range (km), and total trip duration (days). Means of behavioral parameters and track characteristics were compared between clusters using linear models. Tukey contrasts identified significantly different clusters.

#### Foraging patterns compared with environmental conditions

To test whether foraging strategies reflected intrinsic behavioral differences, rather than responses to extrinsic environmental conditions, we compared year, wind strength and lunar phase among population-level clusters (for each species). Years were compared among clusters using chi-squared tests. To compare average wind regimes experienced by birds across clusters, winds were extracted at each location along a track (subsampled to 1 fix every 5 min^−1^) and then a mean was calculated for each bird. Mean winds and mean lunar phase experienced by birds were compared between clusters with ANOVAs and a post hoc Tukey test if clusters were significantly different. Lunar phase was square-root transformed to meet assumptions of normality.

#### Foraging patterns and intrinsic factors

To explore potential drivers of foraging strategies we examined sex, body size, and body condition among birds of different clusters. A chi-squared test tested for sex differences while generalized least squares models tested for differences among clusters in body size and body condition indices.

## Results

Black-footed and Laysan albatrosses predominantly foraged north and northeast of Tern Island in pelagic waters, with a subset of birds from each species (*n* = 6 (30 %) black-footed; *n* = 5 (28 %) Laysan) visiting the sharp bathymetric slope of the northwestern Hawaiian island seamount chain (Additional file 3: Figure S3). Additionally, individuals from both species (*n* = 2 (10 %) black-footed, *n* = 8 (44 %) Laysan) visited deeper seamounts in pelagic waters along their trips both north and south of the breeding colony. Laysan albatrosses ranged farther north than black-footed albatrosses (Additional file 3: Figure S3, Table 1) however this was predominately driven by a small subset of individuals that ranged much farther than the mean population.

**Table 1:** Track characteristics by species (Mean ± SD) and the effects of species and sex on track characteristics (Parameter coefficient ± SD) for black-footed (BFAL) and Laysan (LAAL) albatrosses

	Summary statistics		Linear model output								
	BFAL	LAAL	Species			Sex [BFAL]			Sex [LAAL]		
	Mean ± SD	Mean ± SD	Coeff. ± SD	t	P	Coeff. ± SD	t	P	Coeff. ± SD	t	P
Total distance (km)	886.1 ± 476.9	918.6 ± 496.6	686.1 ± 363.9*	1.9	0.07	402.2 ± 210.1	1.9	0.23	480.9 ± 351.5	−1.368	0.52
Daily travel (km/day)	468.2 ± 176.4	499.2 ± 163.6	198.4 ± 92.2**	2.2	0.04	73.6 ± 74.1	1.0	0.75	186.8 ± 86.4	−2.161	0.15
Max. distance (km)	368.4 ± 215.3	500.2 ± 365.8	11.9 ± 3.4**	3.6	0.001	3.7 ± 2.7	1.4	0.53	10.2 ± 3.1**	−3.240	0.01
Trip duration (days)	1.9 ± 0.65	2.0 ± 0.5	1.5 ± 0.4**	4.2	<0.001	0.5 ± 0.3	1.7	0.32	1.2 ± 0.3**	−3.580	0.01

Significance was set to P ≤ 0.05 (**) and marginal significance to P ≤ 0.1 (*)

### Individual characteristics

**Fig. 3:**
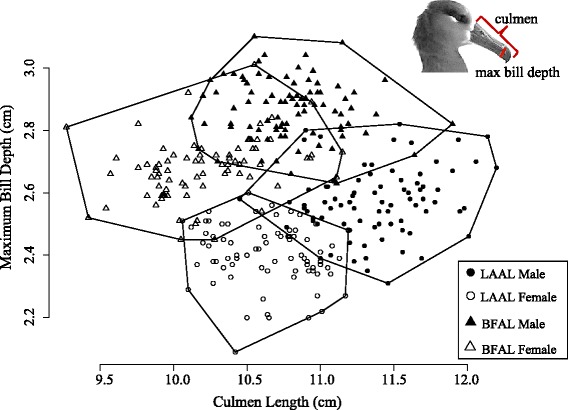
Scatterplot of bill morphometrics indicates clustering in morphological space. Morphometrics cluster by species (shorter, thicker bills in black-footed albatrosses (BFAL)) and by sex within species (female bills generally shorter and less deep). Bill measurements between the sexes overlap more in black-footed albatrosses than they do in Laysan albatrosses (LAAL)

As expected, black-footed albatrosses were slightly heavier in mass than Laysan albatrosses, and within each species, females weighed less than males (Additional file 4: Table S1). Despite their larger mass and overall size, black-footed albatrosses had shorter culmen lengths but thicker bill depth than Laysan albatrosses (Additional file 4: Table S1). Culmen length versus bill depth showed a distinct clustering between species and between sexes (Fig. 3). Black-footed albatross had a shorter range of body size indices, ranging from −2.57 to 1.68, compared to −1.87 to 3.61 for Laysan albatrosses. Black-footed albatrosses had greater overlap in bill morphometrics between the sexes than Laysan albatrosses (Fig. 3). Body size accounted for 20 % of the variation in black-footed albatross adult body mass (F_1,166_ = 42.6, *P* < 0.001) and only 6.2 % of the variation in adult Laysan albatross body mass (F_1,161_ = 11.2, *P* < 0.001).

#### Species and diurnal differences in movement parameters

Movement parameters did not differ between species until examined separately between day and night, with the exception of drift duration (Table 2, Additional file 5: Table S2). Both species spent a similar proportion of total trip in flight, but while time in flight was similar across day and night for black-footed albatross, Laysan albatross spent less of the night in flight. Mean drift duration was overall longer in black-footed albatross than Laysan, and while both species had shorter drifts during the day, this diurnal/nocturnal discrepancy was more pronounced in Laysan albatrosses. There was no difference in mean drift sinuosity between species, and no diurnal differences of mean drift sinuosity within species. Mean landing rates of overall trips were almost identical between the species, but Laysan albatrosses had significantly higher landing rates at night than black-footed. There were no significant differences in overall flight speeds or flight angles between the species and both species decreased speeds and increased turn angles at night.

**Table 2:** Movement parameters by species and astronomical state. Means are given ± SD

	Black-footed albatross				Laysan albatross			
Parameter	Overall trip	Day	Night	Twilight	Overall trip	Day	Night	Twilight
Landing rate (landings/h)	2.1 ± 1.4	3.2 ± 2.3	2.0 ± 1.6	1.7 ± 1.5	2.6 ± 1.2	2.8 ± 1.6	3.6 ± 1.8	1.0 ± 0.9
Drift duration (min.)	20.8 ± 11.1	18.8 ± 13.6	20.4 ± 13.4	14.1 ± 10.5	14.3 ± 5.1	9.8 ± 2.6	16.6 ± 8.8	7.7 ± 2.4
Flight speed (km/h)	44.4 ± 3.6	45.6 ± 4.0	42.7 ± 3.8	45.5 ± 3.6	44.4 ± 2.9	46.9 ± 4.0	40.3 ± 3.1	46.1 ± 3.4
Flight turning angle (°)	50.1 ± 7.3	47.0 ± 7.6	53.5 ± 8.4	48.4 ± 8.0	51.0 ± 10.2	48.8 ± 9.3	54.9 ± 13.3	49.3 ± 10.2
Time in flight (%)	67.7 ± 17.0	67.6 ± 19.5	65.2 ± 27.5	83.1 ± 12.2	70.2 ± 14.8	81.9 ± 0.1	55.8 ± 24.0	94.5 ± 5.8

#### Species differences in general track characteristics

All general track characteristics differed between the species (Table 1). Means of daily distance traveled, maximum distance reached, trip duration, and total distance traveled were all greater in Laysan albatrosses (Table 1). Additionally, compared to males, female Laysan albatrosses reached significantly greater maximum distances (female = 767.1 ± 352.4 km, male = 363.3 ± 281.2 km, Table 1) and had significantly longer trip durations (female = 2.4 ± 0.2 days, male = 1.9 ± 0.6 days, Table 1). In contrast, there were no differences between sexes in black-footed albatross (Table 1).

#### Behavioral states – diurnal and species differences

**Fig. 4:**
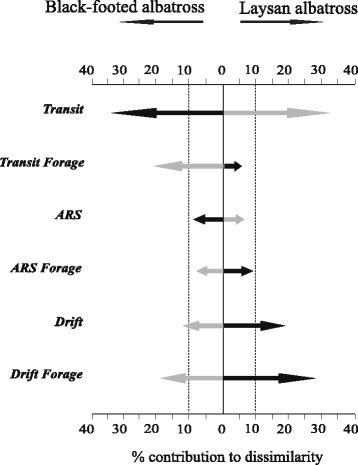
A simper analysis indicates that nocturnal foraging is more prevalent at night in Laysan albatross than it is in black-footed, while the opposite is true for diurnal foraging. Black arrows were calculated on behavior during the night and light grey arrows during the day. The percent contribution to dissimilarity between species is represented by the length of the arrows, and the direction of the arrow is towards the species in which behavior was greatest (measured by duration). Dashed lines at 10 % contribution represent a cutoff below which behavioral states were considered as less important variables driving the dissimilarity between species

The overall behavioral state composition of trips (percent of trip in each behavioral state) between species was not different (*t* = 0.19, *P* = 0.91) with both species spending the largest proportion of trip duration in ‘Transit’, followed by ‘ARS’ and then ‘Drift’ (Table 3). However, when separated into day and night, the behavioral composition of trips were significantly different between species (day: *t* = 3.47, *P* = 0.03 and night: *t* = 2.18, *P* = 0.07). Simper analyses identified which behaviors contributed the most to species dissimilarity (Fig. 4). Percent of trip in ‘Transit Non-Forage’ behavior was the largest contributor to species dissimilarity, contributing 35.6 % in day segments and 32.4 % in night, followed by ‘Drift Forage’ (day: 18.3 %, night: 25.7 %), ‘Drift Non-Forage’ (day:10.4 %, night: 21.9 %) and ‘Transit Forage’ (day: 20.5 %, night: 4.3 %). Both ‘ARS Forage’ and ‘ARS Non-Forage’ contributed <10 % to species dissimilarity, both day and night. All three foraging states (Transit Forage, ARS Forage, and Drift Forage) were greater contributors to species dissimilarity at night in Laysan albatrosses and in day in black-footed albatrosses. On average, both species were in an active foraging mode for almost half their foraging trips (Table 3, 46.4 ± 11.2 %, 44.6 ± 17.6 % for black-footed and Laysan, respectively), however total foraging activity was partitioned between day and night differently for each species (Table 3, 55.8 ± 21.6 % of day and 36.6 ± 21.5 % of night for black-footed albatrosses and 44.6.6 ± 17.6 % of day and 49.3 ± 19.6 % of night for Laysan albatrosses.

**Table 3:** Percentage of time in each behavioral state. Means are given ± SD

	Black-footed albatross			Laysan albatross		
Behavioral state	% Total trip	% Day	% Night	% Total trip	% Day	% Night
Transit non-forage	27.2 ± 7.7	24.3 ± 16.1	31.3 ± 21.9	28.2 ± 7.3	37.7 ± 14.1	17.9 ± 14.2
Transit forage	14.5 ± 10.6	22.6 ± 16.0	6.2 ± 7.4	15.2 ± 7.9	21.8 ± 10.7	8.3 ± 7.6
ARS non-forage	13.9 ± 4.7	7.9 ± 5.6	19.2 ± 12.0	14.0 ± 3.6	10.9 ± 8.2	17.3 ± 8.6
ARS forage	13.8 ± 5.4	17.6 ± 7.0	9.7 ± 9.1	15.0 ± 5.5	15.7 ± 10.7	14.5 ± 9.5
Drift non-forage	10.5 ± 12.7	8.8 ± 10.9	12.1 ± 18.7	8.8 ± 10.2	3.5 ± 6.3	13.8 ± 15.4
Drift forage	18.1 ± 10.6	15.6 ± 14.9	20.6 ± 17.0	16.3 ± 9.2	7.1 ± 5.5	26.5 ± 19.2
Active forage	46.4 ± 11.2	55.8 ± 21.6	36.6 ± 21.5	46.5 ± 7.3	44.6 ± 17.6	49.3 ± 19.6

#### Relationship between lunar phase and behavioral states of albatrosses

**Fig. 5:**
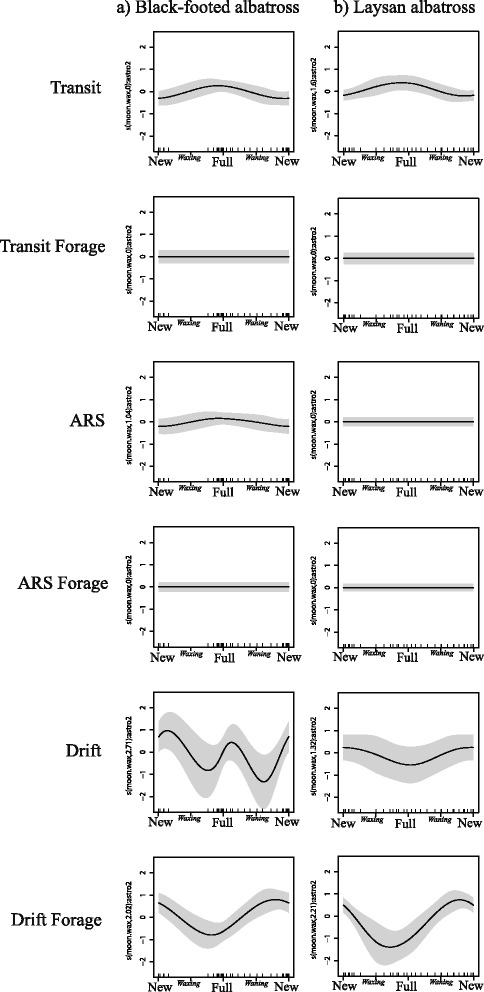
Effects of lunar phase on each behavioral state in a) black-footed and b) Laysan albatrosses. Lunar effect isolated for night portions of trips only. Shaded areas represent 95 % Bayesian confidence intervals [119]

Behavioral states associated with drifting on the water had the greatest response to lunar phase for both species. Black-footed and Laysan albatrosses both increased ‘Drift Forage’ behavior on the darkest nights (Fig. 5, black-footed: *F* = 7.30, *P* < 0.001; Laysan: *F* = 7.48, *P* < 0.001). Both species also increased ‘Drift Non-Forage’ behavior on the darkest nights. However, this was a weak response in Laysan albatrosses (Fig. 5, *F* = 1.17, *P* = 0.06), and black-footed albatrosses also increased ‘Drift Non-Forage’ on the brightest nights (Fig. 5, *F* = 5.12, *P* < 0.001). ‘Transit Non-Forage’ behavior increased with the moon phase for both black-footed and Laysan albatrosses (Fig. 5, *F* = 2.99, *P* = 0.002 and *F* = 2.49, *P* = 0.007, respectively). None of the other flight modes were significantly affected by moon phase, although black-footed albatrosses showed a weak increase in ‘ARS Non-Forage’ during the full moon (Fig. 5, *F* = 0.96, *P* = 0.07).

#### Within species foraging patterns

**Fig. 6:**
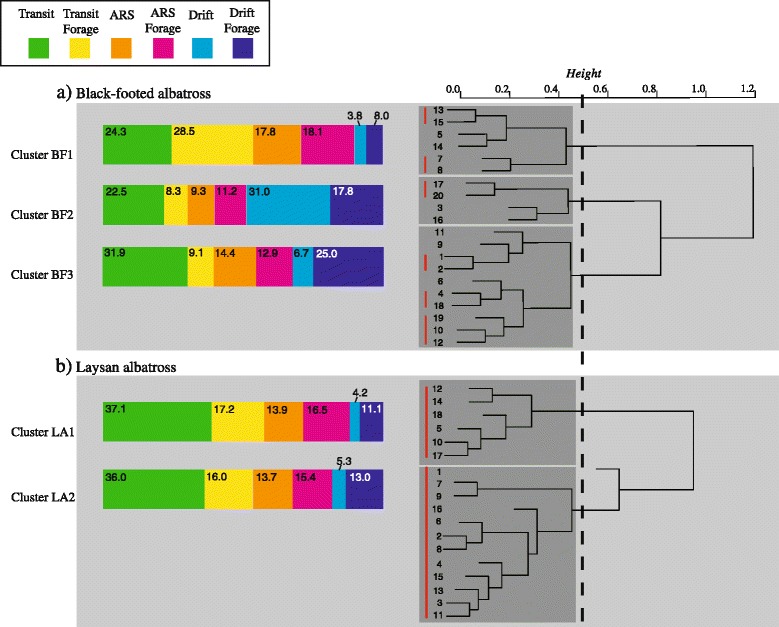
‘Pvclust’ clustering output with the corresponding population-level foraging patterns shows higher population variability for (a) black-footed albatrosses than for (b) Laysan albatrosses. The 50 % similarity level is indicated with the dotted black line providing the threshold for population-level foraging patterns (dark grey shaded rectangles). Clusters significant at the *P* > 0.95 significance level are indicated with the vertical red lines. The mean behavioral composition of trips of birds in each cluster are given as colored bar diagrams with normalized means of each behavioral state (% trip)

The ‘pvclust*’* hierarchical clustering algorithm identified three times as many behavioral clusters in black-footed (six clusters) than Laysan albatrosses (two clusters) at the *P* > 0.95 significance level (indicated by red vertical lines, Fig. 6). Defining population-level foraging patterns at the 50 % similarity level resulted in three behavioral clusters in black-footed and two in Laysan albatrosses (dark grey shaded boxes, Fig. 6) which we used in subsequent analyses.

#### Foraging patterns of black-footed albatrosses

Cluster 1 – BF1 (Fig. 6, Table 4): Individuals from Cluster 1 (BF1) spent the majority of their foraging trips in flight. Of all black-footed albatrosses, birds in BF1 allocated the most foraging time to the ‘Transit Forage’ behavioral state, which predominantly occurred during the day; although compared to birds from BF2 and BF3, they also spent the largest proportion of the night in this foraging mode. Birds from BF1 also spent the largest proportion of night in ‘Transit Non-Forage’. This cluster had the highest percent trip in both the ‘ARS Non-Forage’ and ‘ARS Forage’ behavioral states. Most of the ‘ARS Non-Forage’ behavior occurred during the night while ‘ARS Forage’ was allocated equally between day and night. Birds from BF1 spent very little of their foraging trips drifting on the surface of the water, and both the ‘Drift Non-Forage’ and Drift Forage’ behavioral states occurred during the day more than at night. Correspondingly, these birds had the lowest landing rates, both day and night, and had the highest values for all trip distance and duration metrics. Overall, these birds spent more time foraging during the day than at night, and in flight forage mode, rather than drift.

**Table 4:** Black-footed albatross: Mean values of behavioral parameters and track characteristics for each cluster

				Cluster BF1	Cluster BF2	Cluster BF3
				*N = 6*	*N = 4*	*N = 10*
	*Test*	*F*	*P*	*Mean ± SD*	*Mean ± SD*	*Mean ± SD*
*Behavioral parameters*						
% Day in transit^a^	gls	9.8	0.001**	11.6 ± 1.9	28.9 ± 13.3^c^	30.1 ± 18.3^c^
% Night in transit	lm	3.0	0.08*	36.3 ± 16.7^c^	11.9 ± 8.9	36.0 ± 25.0^c^
% Day in transit forage^b^	lm	12.7	0.0004**	43.0 ± 7.6	12.3 ± 2.6^c^	14.4 ± 10.5^c^
% Night in transit forage^a^	gls	5.8	0.01**	13.7 ± 9.8	2.2 ± 2.6^c^	3.4 ± 2.6^c^
% Day in ARS^b^	lm	0.7	0.51	6.3 ± 6.6	10.5 ± 4.8	7.8 ± 5.4
% Night in ARS^b^	gls	5.1	0.02**	27.1 ± 12.4^c^	7.8 ± 5.2	19.1 ± 10.4^c^
% Day in ARS forage	lm	0.5	0.62	20.0 ± 7.9	15.9 ± 6.9	16.9 ± 6.9
% Night in ARS forage^a^	gls	2.0	0.17	15.0 ± 12.2	5.0 ± 4.7	8.3 ± 7.3
% Day in drift	gls	2.7	0.10*	5.5 ± 6.2^c^	18.4 ± 16.3	7.0 ± 9.3^c^
% Night in drift^a^	gls	18.5	.00001**	2.0 ± 2.8^c^	47.1 ± 9.3	4.2 ± 5.0^c^
% Day in drift forage^b^	lm	0.8	0.47	10.2 ± 5.3	11.5 ± 12.8	20.5 ± 18.6
% Night in drift forage	lm	5.0	0.02**	5.3 ± 6.6	25.2 ± 14.0^c^	28.0 ± 17.1^c^
Day landing rate^a^	lm	0.3	0.75	1.37 ± 0.39	1.7 ± 0.74	1.76 ± 1.03
Night landing rate^b^	lm	2.7	0.10*	0.47 ± 0.28^c^	1.60 ± 1.43^c^	1.04 ± 0.49^c^
*Track characteristics*						
Daily travel (km)	gls	9.9	0.001**	650 ± 115	337 ± 135^c^	412 ± 139^c^
Max range^b^ (km)	gls	2.8	0.09*	551 ± 284	254 ± 107^c^	305 ± 129^c^
Total distance (km)	gls	6.5	0.008**	1352 ± 520	565 ± 140^c^	735 ± 328^c^
Trip duration (km)	lm	0.35	0.71	2.04 ± 0.667	1.77 ± 0.485	1.75 ± 0.742

A linear model (lm) was used when homogeneity was not violated and a generalized least squares model (gls) was applied when parameters showed heterogeneity, incorporating a ‘varIdent’ variance structure

Significance was set at *P* ≤ 0.05 (**) and marginal significance to *P* ≤ 0.1 (*)

^a^log_10_-transformed for gls or lm

^b^square-root transformed for gls or lm

^c^indicates which clusters did not significantly differ from each other at *P* ≤ 0.05 in a multiple comparison post-hoc Tukey test

Cluster 2 – BF2 (Fig. 6, Table 4): Individuals from Cluster 2 (BF2) spent the majority of their foraging trips in on the water. These birds had the lowest allocation of time to either flight foraging mode, and predominantly foraged in the ‘Drift Forage’ state, mostly during the night. BF2 birds had a very large percentage of trip in ‘Drift Non-Forage’, also mostly during the night, but they also had the largest percentage of the day in ‘Drift Non-Forage’ behavior, compared to the other clusters. All four flight behaviors (Transit Forage/ Non-Forage, ARS Forage/ Non-Forage) occurred more during the day than at night. These individuals had the highest landing rates at night and had the lowest values for all trip distance and duration metrics.

Cluster 3 – BF3 (Fig. 6, Table 4): Cluster 3 (BF3) birds relied on both flight and drift behavior. While BF3 birds spent a significant amount of the trip in flight, they predominantly foraged using the ‘Drift Forage’ behavioral state. Although overall time on water was lower than for BF2 birds, they had a higher proportion of the trip in ‘Drift Forage’ mode, and while ‘Drift Forage’ occurred more frequently at night than day within BF3 birds, these birds spent a larger proportion of the day in ‘Drift Forage’ when compared to birds from other clusters. Overall, birds from BF3 allocated the most time to non-foraging flight behaviors compared to the other clusters, but despite the prevalence of flight, they foraged predominantly while drifting, both day and night.

#### Foraging patterns of Laysan albatrosses

Cluster 1 – LA1 and Cluster 2 – LA2 (Fig. 6, Table 5): Both Laysan albatross clusters spent, on average, about one third of their foraging trips in the ‘Transit Non-Forage’ behavioral state, but for LA2 birds, this predominantly occurred during the day, while LA1 birds spent equal amounts of day and night in ‘Transit Non-Forage’. Although both clusters had similar overall behavioral composition of trips, LA1 birds spent comparatively more time foraging in flight, while LA2 birds spent more time drift foraging than LA1 birds. ‘Transit Forage’ at night was more important for LA1 birds, but ‘ARS Forage’ at night was similarly important for both clusters. Overall, LA2 birds spent the majority of the day in ‘Transit Non-Forage’ while spending the majority of the night in ‘Drift Non-Forage’ and especially ‘Drift Forage’, while LA1 birds spent the majority of the day in ‘Transit Forage’ and ‘ARS Forage’ and the majority of the night in ‘Transit Non-Forage’ and ‘ARS Non- Forage’. LA1 and LA2 birds had similar landing rates during the day, but landing rates at night were higher in LA2 birds. Birds from LA1 had greater distance and duration metrics.

**Table 5:** Laysan albatross: Mean values of behavioral parameters and track characteristics for each cluster

				Cluster LA1	Cluster LA2
				*N = 6*	*N = 12*
	*Test*	*F*	*P*	*Mean ± SD*	*Mean ± SD*
*Behavioral parameters*					
% Day in transit	lm	9.2	0.008**	24.7 ± 8.6	43.1 ± 12.3
% Night in transit^b^	lm	4.4	0.05**	28.4 ± 15.1	13.6 ± 11.9
% Day in transit forage	lm	17.2	0.0009**	33.6 ± 7.4	16.9 ± 7.6
% Night in transit forage^b^	lm	7.3	0.02**	15.0 ± 9.7	5.5 ± 4.6
% Day in ARS	lm	4.9	0.04**	4.8 ± 6.8	13.4 ± 7.5
% Night in ARS	lm	5.8	0.03**	24.3 ± 6.3	14.4 ± 8.1
% Day in ARS forage	lm	8.6	0.01**	25.5 ± 6.4	11.7 ± 9.5
% Night in ARS forage^b^	lm	0.1	0.74	15.8 ± 5.5	14.0 ± 11.0
% Day in drift^a^	gls	2.7	0.10*	2.7 ± 2.5	3.8 ± 7.4
% Night in drift^a^	gls	18.5	0.00001**	7.0 ± 9.0	16.7 ± 16.9
% Day in drift forage	lm	0.8	0.48	4.9 ± 3.6	8.0 ± 6.1
% Night in drift forage	lm	16.3	0.001**	8.1 ± 8.8	34.2 ± 17.0
Day landing rate^a^	lm	0.81	0.38	1.59 ± 0.43	1.37 ± 0.64
Night landing rate^a^	lm	7.8	0.01**	0.86 ± 0.37	2.12 ± 1.36
*Track characteristics*					
Daily travel	gls	7.6	0.02**	635 ± 124	443 ± 147
Max distance	gls	2.0	0.17	691 ± 355	421 ± 354
Total distance	gls	9.9	0.009**	1425 ± 380	716 ± 385
Trip duration	lm	1.6	0.23	2.57 ± 0.6	2.03 ± 0.9

A linear model (lm) was used when homogeneity was not violated and a generalized least squares model (gls) was applied when parameters showed heterogeneity, incorporating a ‘varIdent’ variance structure

Significance was set at *P* ≤ 0.05 (**) and marginal significance to *P* ≤ 0.1 (*)

^a^log_10_-transformed for gls or lm

^b^square-root transformed for gls or lm

#### Intrinsic factors (Sex, body size and body condition) and foraging patterns (Fig. 7a)

**Fig. 7:**
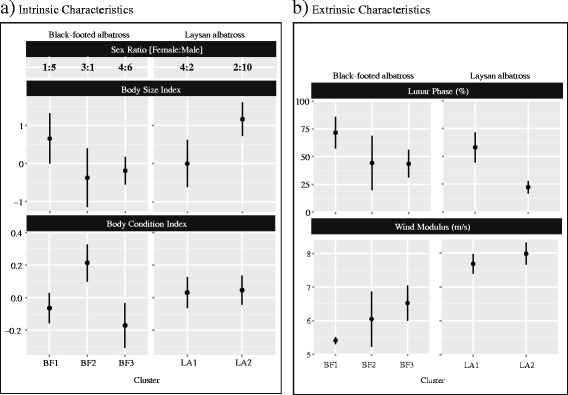
The effect of intrinsic and extrinsic factors on foraging patterns in (a) black-footed and (b) Laysan albatross. Factors are plotted as means ± SE

**Fig. 8:**
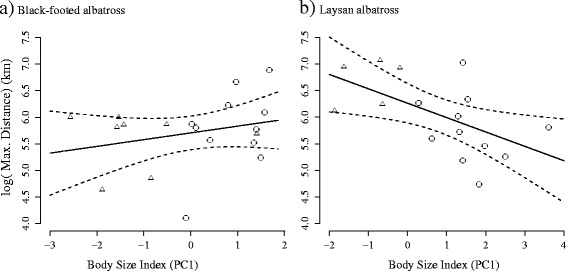
Female Laysan albatrosses reach more distant foraging grounds than males. A linear regression shows no relationship between body size and maximum distance in (a) black-footed albatrosses (F_1,17_ = 1.26, *P* = 0.28, R^2^ = 0.07), whereas maximum distance shows a significant negative correlation with body size in (b) Laysan albatrosses (F_1,14_ = 6.73, *P* = 0.02, *R* ^*2*^ = 0.33). For both species, males are plotted with an open circle and females with an open triangle. Dotted lines represent 95 % confidence intervals

There were neither sex nor body size differences among clusters in black-footed albatrosses (sex: *χ*^2^ = 3.40, *P* = 0.18; body size: F_2,16_ = 0.93, *P* = 0.42); however, a posthoc Tukey HSD test revealed a trend towards higher body condition in birds of Cluster 2, but this was not significant (*t* = 1.38, *P* = 0.19), probably because of the small number of individuals in Cluster 2. Laysan albatrosses did trend towards sex and body size differences between clusters, with the first cluster being composed of smaller birds (F_1,14_ = 2.22, *P* = 0.16) and more females than males (*χ*^2^ = 2.53, *P* = 0.11). Body condition was the same between the two clusters of Laysan albatross (F_1,14_ = 0.01, *P* = 0.92). To explore the potential for competitive exclusion within each species, we used a linear regression to test the effect of body size on maximum distance reached from the colony. There was a significant relationship in Laysan albatrosses, with smaller (and female) individuals reaching farther maximum distances (Fig. 8a, F_1,14_ = 6.73, *P* = 0.02) than larger (and male) individuals. Regressions run separately for the sexes showed different slopes between the sexes (Fig. 8a, −0.18 for male and 0.24 for females), but sample sizes of sexes nested within species were small and sex-specific regressions did not show a significant relationship between body size and maximum distance. There was no relationship with body size and maximum distance reached for black-footed albatrosses (Fig. 8b, F_1,17_ = 1.26, *P* = 0.28) but sex-specific regressions also had different slopes (Fig. 8b, 0.52 for male and 0.0001 for females). Year as a cofactor had no effect for either species and was removed in final regression models.

#### Extrinsic factors (year, wind and lunar phase) and foraging patterns (Fig. 7b)

There was no difference in distribution of years among clusters in both black-footed and Laysan albatrosses (χ-squared = 6.12, P = 0.19 and χ -squared = 5.4, P = 0.15, respectively). Lunar phase was not significantly different between clusters in black-footed albatrosses (F_2,17_ = 1.65, P = 0.22), but wind strength experienced by birds was marginally different between two of the three clusters (F_2,17_ = 2.44, P = 0.12), with birds from Cluster 1 experiencing lower mean winds than Cluster 3 (Tukey’s HSD, P = 0.09). However, flight behaviors were more important in Cluster 1 birds experiencing lower winds (mean wind strength = 5.40 ± 0.25 m/s) than Cluster 3 birds (mean wind strength = 6.52 ± 1.66 m/s), directly contradicting what one would expect if behavioral clusters were being driven by wind speeds alone. Mean wind was not different between the two Laysan albatross clusters (F_1,16_ = 0.30, P = 0.59). Laysan albatrosses in Cluster 1 did experience a fuller lunar phase (52.0 ± 33.4 % disc illuminated) than did birds from Cluster 2 (22.0 ± 20.0 % disc illuminated) (F_1,16_ = 4.01, P = 0.06). However, Cluster 1 birds also flew more during the day than birds from Cluster 2, suggesting the heavy reliance on flight in these birds was not just an artifact of lunar conditions.

## Discussion

### Beyond “area-restricted search” foraging behavior

Many studies classify short duration landings as foraging activity, whereas long drifting bouts that occur at night are interpreted as resting, non-foraging periods. However, drift sinuosity and ingestion events captured by GPS and stomach-temperature data-loggers indicate that these drifts are often associated with active “sit-and-wait” foraging [36, 69, 70, 84, 85]. Although prey consumed in this manner tend to have smaller mass [69, 70], the energetic content of prey available at night (e.g., myctophids and pelagic crustaceans) can be high [86, 87], and the “sit-and-wait” strategy may, at times, be the most optimal strategy (i.e., most energy gained for energy used) [83, 85].

Foraging in direct flight and “sit-and-wait” foraging tactics were as frequently used as area-restricted search flight for brood-guarding Hawaiian albatross, but the use of ARS as the only proxy of foraging behavior is widely prevalent in seabird foraging studies. ARS is an informative metric valuable in identifying areas of high-use and for understanding spatial scales employed by foraging animals. However, ARS is often measured as a two dimensional spatial metric [67] and if the aim is to understand foraging behavior and/or activity budgets, our results caution against only considering ARS behavior, at least for albatrosses and other species that have flexible foraging tactics. Methods like first passage time and residence time are useful in identifying where animals spend the most time, but by themselves do not incorporate behavioral variability within those areas. Using ARS metrics alone, it would be possible to delineate “sit-and-wait” foraging from foraging in flight due to the large differences in spatial scales (10^2^ m vs 10^4^ m) these behaviors operate on; however, it would not be possible to effectively delineate the difference between the two flight-based foraging strategies that operate on similar spatial scales. For example, it is possible to calculate the same value of residence time within a virtual circle for a bird flying in a straight line (direct flight) and landing frequently as for a bird flying in a tortuous path but not landing at all. Therefore, by incorporating a measure of both spatial-temporal use (residence time) and activity (landings), we were able to identify behaviors that would likely be masked if using ARS metrics alone.

Fractal landscape methods that quantify track convolution (i.e., searching intensity) within ARS regions [88, 89] circumvent some limitations of ARS methods that use time as their metric; however, at least for brood-guarding Hawaiian albatrosses, it was quite common for birds to fly in tortuous paths but not land, especially for black-footed albatrosses at night. If we were evaluating the movement of birds solely by identifying path tortuosity without landing activity we could make the erroneous assumption that these birds were actively foraging, e.g., both searching for food *and* landing to feed. In the case of black-footed albatrosses, without considering landing densities within regions of high residency times, we would calculate that they are in ARS flight ~29 % of the night versus ~24 % of the day and might conclude the importance of nocturnal foraging in this species. However, when delineating ARS flight into ‘ARS Forage’ and ‘ARS Non-Forage’ by looking at landing densities, we see that ~19 % of the night is in ‘ARS Non-Forage’ while only 10 % of that is in ‘ARS Forage’, an important distinction that leads us to a different conclusion. Thus, incorporating measurements of activity, such as stomach-temperature loggers [70], accelerometers [90], altimeters [91], wet/dry data [92], etc., within analyses of spatial use is critical for understanding the sometimes nuanced behavior of animals.

### Nocturnal and diurnal niche partitioning between Hawaiian albatross species

Overall foraging behavior was remarkably similar between black-footed and Laysan albatrosses. However, clear species differences emerged when delineating behavior by day and night. Our results support greater nocturnality in Laysan albatrosses at least during the brood-guard. While both species appear to rely on daylight for foraging while in transit, Laysan albatrosses spent more of the night foraging in area-restricted search flight and foraging while drifting than black-footed albatrosses. Black-footed albatrosses appear to rely on daylight for both flight foraging modes, and they also drift foraged in daylight as much as they did at night (Table 3, Fig. 4). Although Laysan albatross foraging behavior suggests greater nocturnality of the two Hawaiian albatrosses, foraging occurred across day and night, to varying degrees, in both species. Foraging trips of Hawaiian albatross were very active (~50 % trip in active forage mode) - likely a reflection of the high provisioning demands on parents during the brood-guard.

Seabirds can adjust their dependency on nocturnal and diurnal foraging in different marine habitats [93] or under conditions of increased competition [36]; the occurrence of nocturnal foraging in Hawaiian albatross, therefore, may be a behavioral response to foraging in a tropical environment during a period of high competition. The oligotrophic waters around the Hawaiian islands are generally described as patchy and nutrient-poor [15], but they do support a large biomass of micronekton associated with steep gradients of the archipelago and nearby seamounts [94, 95]. This micronekton is associated with the diel vertically migrating prey community – the primary prey resource in oceanic waters [94] – that has a more pronounced migration in lower than higher latitudes [12]. Indeed, a recent investigation of stomach contents of fishery by-caught Laysan albatrosses found myctophids to occur more frequently in the stomachs of birds from the Hawaiian fishery compared to the Alaskan fishery [96]. Given the greater abundance of this prey field in surface waters at night in tropical, pelagic waters, nocturnal foraging would provide enhanced feeding opportunities for Hawaiian albatrosses given the relatively long nights (~10–11 h) of the boreal spring. Brood-guarding birds that need to maximize provisioning rates while minimizing trip duration [97] would have an energetic advantage if able to exploit the abundant micronekton in surface waters at night (see [83]), especially considering half the duration of foraging trips occurs at night for brood-guarding Hawaiian albatrosses (48.0 ± 11.7 % and 50.5 ± 4.8 %, for black-footed and Laysan albatrosses, respectively).

How albatrosses search for and locate prey at night is poorly understood; however, foraging by flight in seabirds requires visual cues. The eyes of Laysan albatrosses have high concentrations of rhodopsin, a light sensing pigment (16.30 optical density units (D/g), as compared to 3.90 D/g for black-footed albatrosses and 19.50 D/g for the barn owl, *unpublished data* [40]), indicating a morphological adaptation for higher visual acuity at night. Indeed, foraging in ARS flight remained important at night for Laysan albatrosses – behavior that implies an ability to search and locate prey through visual cues at night. Additionally, nocturnal foraging in flight for Laysan albatrosses was not limited to birds foraging under bright moonlight conditions (cluster 1 birds). While cluster 1 birds did spend more time in transit forage at night, cluster 2 birds, which foraged under darker nocturnal conditions, spent a similar proportion of night actively foraging in area-restricted search behavior as cluster 1 birds. Laysan albatrosses appear to rely less upon moonlight to forage in flight than black-footed albatrosses; however, moonlight likely assists navigation and orientation given the predominance of transit behavior on bright nights (cluster 1 birds, also Fig. 5).

In contrast, black-footed albatross behavior showed a significant reduction of nighttime flight forage behaviors and relied predominately on the “sit-and-wait” strategy at night to forage. Despite a reduction of foraging in flight under dark conditions, black-footed albatrosses spent a large proportion of night on the wing, perhaps commuting to areas where the “sit-and-wait” foraging strategy was profitable. Interestingly, BF1 (cluster 1) birds experienced the brightest moon conditions (Fig. 7b) and were the only cluster of birds within black-footed albatrosses that spent a substantial proportion of the night in both flight foraging strategies, further supporting our conclusion that black-footed albatrosses have a greater reliance on moonlight compared to Laysan albatrosses for flight foraging. Both species showed a strong reduction in drift forage behavior on full moon nights (Fig. 5), likely due to the inaccessibility of diel-migrating prey on bright nights. Increased transit but decreased foraging behavior suggests a reduced foraging efficiency on bright moonlit nights as compared to darker nights as seen in other seabird species [84, 93, 98, 99] but see [100].

### Within- and among- species niche partitioning in a community of albatross

Which mechanisms facilitate coexistence within a community of similar species is a central question in ecology [101]. Intraspecific competition may be more intense than that between species, because smaller morphological differences lead to fewer opportunities for niche partitioning [102]. Indeed, colonies of conspecific seabirds that are in close proximity often have highly delineated foraging grounds reducing intraspecific competitive interactions [46, 103, 104]. Within a colony, density-dependent competition can select for individuals with “roving” strategies [105, 106] – increased range, longer trips, decreased time at resource patches – sometimes with reduced fitness [106]. In Laysan albatrosses, birds from cluster 1 (LA1) traveled more, reached further maximum distances, and spent less time on water (Table 5), consistent with such a “roving” strategy.

Birds from cluster 1 were smaller, and were mostly females, so these differences might reflect sex-specific foraging strategies in brood-guarding Laysan albatrosses. Sex-specific foraging occurs across seabird taxa in dimorphic (see review in [107]), reversed dimorphic [90], and, increasingly, in monomorphic species [107–110]. Compared to black-footed albatrosses, there was greater distance between the sexes in bill dimensions in Laysan albatrosses, suggesting a potential morphological mechanism of intraspecific niche separation. Thus these differences in foraging behavior appear not to be the result of short-term behavioral plasticity but rather fixed trait-mediated niche specialization between the sexes [49], although these morphological differences are very slight compared to other dimorphic albatross species. The longer trip durations and further maximum ranges of female Laysan albatrosses might reflect enhanced flight efficiency of the smaller sex, as is seen in other Procelleriform species that use gliding flight [49, 111]. But if it is flight efficiency driving these behaviors, we would expect to additionally see a relationship between body size and maximum range *within* each sex, and that is not the case (Fig. 8), although sample size are small. Competitive exclusion of smaller females by larger males from foraging grounds near the colony also seems unlikely as there remains significant spatial overlap in core foraging grounds between the sexes.

The prevalence of studies showing sex-specific foraging strategies in monomorphic or slightly sexual dimorphic species suggests sex differences can be unrelated to body size [108, 109, 112]. An alternative explanation is that the foraging differences between the sexes in Laysan albatrosses are not related to size but to different parental roles, with shorter trips of males reflecting male-biased provisioning. During incubation, male Laysan albatrosses spend more time incubating the egg than females who spend more time foraging at sea [113], likely regaining body condition lost in egg production [25]. It is possible that male-biased nest attendance continues into the brood-guard stage as females continue to allocate more energy to self-maintenance than males. Parental roles can switch across the breeding season with one sex contributing more time and energy to the nest early in season, and the other sex contributing more later in season [108, 109], so it would be informative to conduct a study of fine-scale foraging behavior across the breeding season to see if sex-specific differences persist into the post-guard stage.

The breeding population of black-footed albatrosses in the French Frigate Shoals (Tern Island and surrounding atolls) is ~30 % larger than that of Laysan albatrosses [114]. Despite larger numbers of breeding birds, black-footed albatrosses have shorter maximum ranges and trip durations than Laysan albatrosses (Table 1). We can thus expect higher densities of black-footed albatrosses at-sea that should result in greater intra-specific competition in foraging grounds near Tern Island. Often, a high level of individual variability unrelated to morphological traits is a flexible behavioral response to increased intraspecific competition [55, 56, 115]. Therefore, it is not surprising we see greater levels of variability in foraging strategies in black-footed albatrosses, independent of body size or sex. Conducting a similar study at a different breeding colony, such as Midway Atoll, where densities of Laysan albatrosses are greater than that of black-footed albatrosses would help to clarify whether intraspecific variability in these species is a short-term behavioral response dictated by density of conspecifics or is a fixed intrinsic characteristic of the species.

Greater population-level behavioral plasticity in black-footed albatrosses might serve as a buffer against environmental variability on breeding decisions. Indeed, black-footed albatrosses breeding at Tern Island show more behavioral flexibility in response to poor environmental conditions than Laysan albatrosses do and have higher reproductive success at Tern Island in “poor” years [116]. Furthermore, different behavioral patterns within black-footed albatrosses appear to have varying efficiencies, at least for cluster 2 birds (BF2), although the higher mean body condition of these birds was not statistically significant (Fig. 7a). However, it is interesting that the birds with higher mean body condition were the birds that predominantly foraged while drifting and spent a much larger proportion of their foraging trips on the water. Foraging on the water in “sit-and-wait” was found to be the most energy efficient foraging strategy in a study of wandering albatrosses during the brood-guard [83]. Variable efficiencies of foraging patterns are likely to have a more measurable effect on individuals in the brood-guard when constraints are high [117] and when birds are foraging in challenging environmental conditions [118].

## Conclusion

We quantified behavioral mechanisms that enable coexistence within an albatross community constrained to nutrient-poor tropical waters during the most energetically-demanding reproductive stage in birds: the brood-guard. Albatrosses showed discrete behavioral partitioning both between and within species which was driven by differences in nocturnal and diurnal foraging and by sex-specific strategies. Our results are the first to observe nocturnally-biased foraging behavior in Laysan albatrosses, but we emphasize foraging behavior occurred across day and night in both species. Black-footed albatrosses exhibited greater variability in foraging patterns suggesting they may experience strong intraspecific competition at Tern Island. While foraging patterns in Laysan albatrosses were less variable, behavioral differences were primarily associated with sex. Examining variability in fine-scale foraging behavior across breeding phases, when birds experience large shifts in oceanic habitat and levels of competition will provide further understanding of behavioral plasticity and capacity for short-term adaptation in Hawaiian albatross.

## Additional files

Additional file 1: Figure S1. A bimodal distribution of speeds in both species, indicating a speed threshold of ~6 km/h below which birds do not remain aloft. (PDF 338 kb) Additional file 2: Figure S2. Wind speed did not affect sinuosity of tracks in drifting birds, validating the use of drift sinuosity as a proxy for drift foraging activity. Wind speeds were extracted at the first location of every drift, so that each drift had an associated wind speed value. A linear mixed model was constructed with drift sinuosity as a continuous response variable to wind speed with species as a fixed factor. Individual bird was included as a random effect since birds had numerous drifts within a foraging trip. Slopes of individual birds were allowed to vary and are represented by the thinner lines, while the population mean slope (of both species) is the bold line. Wind speed, nor species, had a significant effect on sinuosity (t_35,655_ = −1.11, P = 0.27). (PDF 124 kb) Additional file 3: Figure S3. Black-footed (black) and Laysan (white) albatross GPS tracks deployed at Tern Island (black star) during the brood-guard stage in 2006, 2009, 2010 and 2012. Tracks were subsampled to 1 location every 5 min for the purpose of this illustration. Some individuals of both species visited both the steep bathymetric slope of the northwestern Hawaiian Islands as well as deeper submerged seamounts in pelagic waters. Both species predominately foraged north and northeast of Tern Island with a few individuals from both species foraging south and southwest. The vast majority of trip durations were spent in pelagic waters. (PDF 5217 kb) Additional file 4: Table S1. Mass and morphometrics (means ± SD) of Laysan and black-footed albatross breeding on Tern Island. (DOCX 13 kb) Additional file 5: Table S2. Effects of species, astronomical state and their interaction on movement parameters (Parameters coefficient ± SD). (XLSX 12 kb)

## Abbreviations

LAAL: Laysan albatross
BFAL: Black-footed albatross

## References

[1] Lack DL (1968). Ecological adaptations for breeding in birds.

[2] Stearns SC (1992). *The evolution of life histories. Volume 249*.

[3] Owens IPF, Bennett PM (1995). Ancient ecological diversification explains life-history variation among living birds. Proc R Soc London B Biol Sci.

[4] Martin TE. Food as a limit on breeding birds. A life-history perspective. Annu Rev Ecol Syst. 1987;18:453–87.

[5] Weimerskirch H. Seabird Demography and Its Relationship with the Marine Environment. In Biology of Marine Birds. CRC Press; Boca Raton, Florida. 2001:115–136.

[6] Ricklefs RE (1990). Ecology.

[7] Crossin GT, Phillips RA, Wynne-Edwards KE, Williams TD. Post-migratory body condition and ovarian steroid production predict breeding decisions by female gray-headed albatrosses. Physiol Biochem Zool. 2013;86:761–8. 10.1086/673755 24241072

[8] Chastel O, Weimerskirch H, Jouventin P (1995). Influence of body condition on reproductive decision and reproductive success in the blue petrel. Auk.

[9] Perrins CM (1970). The timing of birds’ breeding seasons. Ibis.

[10] Le Corre M (2001). Breeding seasons of seabirds at Europa Island (southern Mozambique Channel) in relation to seasonal changes in the marine environment. J Zool.

[11] Bertram DF, Mackas DL, McKinnell SM (2001). The seasonal cycle revisited: interannual variation and ecosystem consequences. Prog Oceanogr.

[12] Ashmole NP (1971). Seabird ecology and the marine environment. Avian Biol.

[13] Nelson JB (1983). Contrasts in breeding strategies between some tropical and temperate marine pelecaniformes. Stud Avian Biol.

[14] Ainley DG, Llano G (1977). Feeding methods in seabirds: a comparison of polar and tropical nesting communities in the eastern Pacific ocean. Adaptations within Antarctic ecosystems. Proceedings of the Third SCAR Symposium on Antarctic Biology.

[15] Seki M, Polovina J (2001). Biological enhancement at cyclonic eddies tracked with GOES thermal imagery in Hawaiian waters. Geophys Res Lett.

[16] Haney J: Seabird affinities for Gulf Stream frontal eddies: responses of mobile marine consumers to episodic upwelling. J Mar Res. 1986:361–84.

[17] Haney JC (1986). Seabird Patchiness in Tropical Oceanic Waters: The Influence of Sargassum “Reefs.”. Auk.

[18] Tew Kai E, Rossi V, Sudre J, Weimerskirch H, Lopez C, Hernandez-Garcia E, et al. Top marine predators track Lagrangian coherent structures. Proc Natl Acad Sci. 2009;106(20):8245–50. 10.1073/pnas.0811034106 PMC2677090 19416811

[19] Harrison CS (1990). Seabirds of Hawaii: natural history and conservation.

[20] Harris MP (1969). Breeding seasons of sea-birds in the Galapagos Islands. J Zool.

[21] Schreiber RW, Ashmole NP (1970). Sea-bird breeding seasons on Christmas Island, Pacific Ocean. Ibis.

[22] Ballance LT, Pitman RL. S34 . 4. Foraging ecology of tropical seabirds. 1999.

[23] Hyrenbach K, Fernández P, Anderson D (2002). Oceanographic habitats of two sympatric North Pacific albatrosses during the breeding season. Mar Ecol Prog Ser.

[24] Kappes MA. Comparative foraging ecology and energetics of albatrosses. Dissertation from University of California, Santa Cruz. 2009.

[25] Tickell WLN. Albatrosses. Yale University Press. 2000.

[26] Drent RH, Daan S (1980). The prudent parent: energetic adjustments in avian breeding. Ardea.

[27] Ricklefs R, Johnston R (1983). Comparative avian demography. *Current Ornithology SE - 1. Volume 1*.

[28] Weimerskirch H, Lys P (2000). Seasonal changes in the provisioning behaviour and mass of male and female wandering albatrosses in relation to the growth of their chick. Polar Biol.

[29] Birt VL, Birt TP, Goulet D, Cairns DK (1987). Ashmole’s halo: direct evidence for prey depletion by a seabird. Mar Ecol Prog Ser.

[30] Ashmole NP (1963). The regulation of numbers of tropical oceanic birds. Ibis.

[31] Schreiber EA, Burger J: Biology of Marine Birds. CRC Press; Boca Raton, Florida. 2001.

[32] Fernández P, Anderson DJ, Sievert PR, Huyvaert KP (2001). Foraging destinations of three low‐latitude albatross (Phoebastria) species. J Zool.

[33] Kappes MA, Shaffer SA, Tremblay Y, Foley DG, Palacios DM, Robinson PW, et al. Hawaiian albatrosses track interannual variability of marine habitats in the North Pacific. Prog Oceanogr. 2010;86:246–60.

[34] Fischer KN, Suryan RM, Roby DD, Balogh GR (2009). Post-breeding season distribution of black-footed and Laysan albatrosses satellite-tagged in Alaska: Inter-specific differences in spatial overlap with North Pacific fisheries. Biol Conserv.

[35] Gutowsky SE, Gutowsky L, Jonsen ID, Leonard ML, Naughton MB, Romano MD, et al. Daily activity budgets reveal a quasi-flightless stage during non-breeding in Hawaiian albatrosses. Mov Ecol. 2014;2:23. 10.1186/s40462-014-0023-4 PMC4337467 25709832

[36] Zavalaga CB, Dell’Omo G, Becciu P, Yoda K (2011). Patterns of GPS Tracks Suggest Nocturnal Foraging by Incubating Peruvian Pelicans (Pelecanus thagus). PLoS One.

[37] Ballance LT, Pitman RL, Reilly SB (1997). Seabird community structure along a productivity gradient: importance of competition and energetic constraint. Ecology.

[38] González-Solís J, Croxall JP, Wood AG (2000). Sexual dimorphism and sexual segregation in foraging strategies of northern giant petrels, Macronectes halli, during incubation. Oikos.

[39] Harrison CS, Hida TS, Seki MP (1983). Hawaiian Seabird Feeding Ecology. Wildl Monogr.

[40] Harrison CS, Sillman AJ. Personal communication. Univ. California, Davis. 2012.

[41] Fernández P, Anderson DJ (2000). Nocturnal and diurnal foraging activity of Hawaiian albatrosses detected with a new immersion monitor. Condor.

[42] Pitman RL, Walker WA, Everett WT, Gallo-Reynoso JP (2004). Population status, foods and foraging of Laysan albatrosses Phoebastria immutabilis nesting on Guadalupe Island, Mexico. Mar Ornithol.

[43] Walker WA, Pitman RL, Ballance LT. Wanted: Dead or Alive? Hawaiian Albarosses Feed Mainly by Scavenging on Mesopelagic Cephalopods. Poster presented at the 40^th^ Annual Pacific Seabird Group Meeting. 2013.

[44] MacArthur RH (1958). Population ecology of some warblers of northeastern coniferous forests. Ecology.

[45] Villegas-Amtmann S, Costa DP, Tremblay Y, Salazar S, Aurioles-Gamboa D (2008). Multiple foraging strategies in a marine apex predator, the Galapagos sea lion Zalophus wollebaeki. Mar Ecol Ser.

[46] Masello JF, Mundry R, Poisbleau M, Demongin L, Voigt CC, Wikelski M, et al. Diving seabirds share foraging space and time within and among species. Ecosphere. 2010;1:art19.

[47] Araújo MS, Bolnick DI, Machado G, Giaretta AA, dos Reis SF (2007). Using delta13C stable isotopes to quantify individual-level diet variation. Oecologia.

[48] Jeglinski JWE, Goetz KT, Werner C, Costa DP, Trillmich F (2013). Same size - same niche? Foraging niche separation between sympatric juvenile Galapagos sea lions and adult Galapagos fur seals. J Anim Ecol.

[49] Shaffer SA, Weimerskirch H, Costa DP (2001). Functional significance of sexual dimorphism in wandering albatrosses, Diomedea exulans. Funct Ecol.

[50] Polis GA (1984). Age Structure Component of Niche Width and Intraspecific Resource Partitioning: Can Age Groups Function as Ecological Species?. Am Nat.

[51] Patrick SC, Weimerskirch H (2014). Personality, foraging and fitness consequences in a long lived seabird. PLoS One.

[52] Patrick SC, Weimerskirch H (2014). Consistency pays: sex differences and fitness consequences of behavioural specialization in a wide-ranging seabird. Biol Lett.

[53] Watanuki Y (1992). Individual diet difference, parental care and reproductive success in slaty-backed gulls. Condor.

[54] Votier SC, Bearhop S, Ratcliffe N, Furness RW (2004). Reproductive consequences for great skuas specializing as seabird predators. Condor.

[55] Svanbäck R, Bolnick DI (2007). Intraspecific competition drives increased resource use diversity within a natural population. Proc Biol Sci.

[56] Tinker MT, Bentall G, Estes JA (2008). Food limitation leads to behavioral diversification and dietary specialization in sea otters. Proc Natl Acad Sci.

[57] Araújo MS, Bolnick DI, Layman CA (2011). The ecological causes of individual specialisation. Ecol Lett.

[58] Nevitt GA, Losekoot M, Weimerskirch H (2008). Evidence for olfactory search in wandering albatross, Diomedea exulans. Proc Natl Acad Sci.

[59] Suryan RM, Anderson DJ, Shaffer SA, Roby DD, Tremblay Y, Costa DP, et al. Wind, waves, and wing loading: morphological specialization may limit range expansion of endangered albatrosses. PLoS One. 2008;3:e4016. 10.1371/journal.pone.0004016 PMC2602987 19107200

[60] Flint E. Hawaiian islands national wildlife refuge and midway atoll national wildlife refuge – annual nest counts through hatch year 2009. U.S. Fish and Wildlife Service, unpublished report. 2009.

[61] IUCN 2014. The IUCN Red List of Threatened Species [http://www.iucnredlist.org].

[62] Véran S, Gimenez O, Flint E, Kendall WL, Doherty JRPF, Lebreton J-D (2007). Quantifying the impact of longline fisheries on adult survival in the black-footed albatross. J Appl Ecol.

[63] Lewison RL, Crowder LB (2003). Estimating fishery bycatch and effects on a vulnerable seabird population. Ecol Appl.

[64] Fritz H, Said S, Weimerskirch H (2003). Scale-dependent hierarchical adjustments of movement patterns in a long-range foraging seabird. Proc R Soc B Biol Sci.

[65] Phillips RA, Xavier JC, Croxall JP, Burger AE (2003). Effects of satellite transmitters on albatrosses and petrels. Auk.

[66] Vandenabeele SP, Shepard EL, Grogan A, Wilson RP (2011). When three per cent may not be three per cent; device-equipped seabirds experience variable flight constraints. Mar Biol.

[67] Fauchald P, Tveraa T (2003). Using first-passage time in the analysis of area-restricted search and habitat selection. Ecology.

[68] Kareiva P, Odell G. Swarms of predators exhibit “prey-taxis” if individual predators Use area-restricted search. Am Nat. 1987;130:233–70.

[69] Weimerskirch H, Pinaud D, Pawlowski F, Bost CA. Does prey capture induce area‐restricted search? A fine‐scale study using GPS in a marine predator, the wandering albatross. Am Nat. 2007;170:734–43. 10.1086/522059 17926295

[70] Catry P, Phillips RA, Phalan B, Silk JRD, Croxall JP. Foraging strategies of grey-headed albatrosses Thalassarche chrysostoma: integration of movements, activity and feeding events. Mar Ecol Prog Ser. 2004;280:261-73.

[71] Harper PC (1987). Feeding behavior and other notes on 20 species of Procelleriformes at sea. Notornis.

[72] Barraquand F, Benhamou S (2008). Animal movements in heterogeneous landscapes: identifying profitable places and homogeneous movement bouts. Ecology.

[73] Pearson RK: Mining Imperfect Data: Dealing with Contamination and Incomplete Records. Siam; Philadelphia, Pennsylvania. 2005.

[74] Bograd SJ, Rabinovich AB, LeBlond PH, Shore JA (1997). Observations of seamount-attached eddies in the North Pacific. J Geophys Res.

[75] Chaigneau A, Pizarro O, Rojas W (2008). Global climatology of near-inertial current characteristics from Lagrangian observations. Geophys Res Lett.

[76] Weimerskirch H, Guionnet T, Martin J, Shaffer SA, Costa DP (2000). Fast and fuel efficient? Optimal use of wind by flying albatrosses. Proc R Soc B Biol Sci.

[77] Pinheiro J, Bates D, DebRoy S, Sarkar D, R Core Team (2015). Nlme: linear and nonlinear mixed effects models.

[78] Wood SN (2011). Fast stable restricted maximum likelihood and marginal likelihood estimation of semiparametric generalized linear models. J R Stat Soc Ser B Statistical Methodol.

[79] Hothorn T, Bretz F, Westfall P (2008). Simultaneous inference in general parametric models. Biometrical J.

[80] Suzuki R, Shimodaira H (2006). Pvclust: an R package for assessing the uncertainty in hierarchical clustering. Bioinforma.

[81] R Core Team (2015). R: A language and environment for statistical computing. R Foundation for Statistical Computing, Vienna,Austria. http://www.R-project.org/.

[82] Clarke KR (1993). Non‐parametric multivariate analyses of changes in community structure. Aust J Ecol.

[83] Louzao M, Wiegand T, Bartumeus F, Weimerskirch H (2014). Coupling instantaneous energy-budget models and behavioural mode analysis to estimate optimal foraging strategy: an example with wandering albatrosses. Mov Ecol.

[84] Cruz SM, Hooten M, Huyvaert KP, Proaño CB, Anderson DJ, Afanasyev V, et al. At-sea behavior varies with lunar phase in a nocturnal pelagic seabird, the swallow-tailed gull. PLoS One. 2013;8:e56889. 10.1371/journal.pone.0056889 PMC3582633 23468889

[85] Jodice PGR, Roby DD, Suryan RM, Irons DB, Kaufman AM, Turco KR, et al. Variation in energy expenditure among black-legged kittiwakes: effects of activity-specific metabolic rates and activity budgets. Physiol Biochem Zool. 2003;76:375–88. 10.1086/375431 12905124

[86] Roby DD, Turco KR, Anthony JA (1997). Diet Composition, reproductive energetics, and productivity of seabirds damaged by the Exxon Valdez Oil spill. Restoration Project 97163 G Annual Report. Oregon Cooperative Fish and Wildlife Research Unit.

[87] Paredes R, Orben RA, Suryan RM, Irons DB, Roby DD, Harding AMA, et al. Foraging responses of black-legged kittiwakes to prolonged food-shortages around colonies on the Bering Sea shelf. PLoS One. 2014;9:e92520. 10.1371/journal.pone.0092520 PMC3966792 24671108

[88] Tremblay Y, Roberts AJ, Costa DP (2007). Fractal landscape method: an alternative approach to measuring area-restricted searching behavior. J Exp Biol.

[89] Nams V (1996). The VFractal: a new estimator for fractal dimension of animal movement paths. Landsc Ecol.

[90] Weimerskirch H, Corre M, Ropert-Coudert Y, Kato A, Marsac F (2006). Sex-specific foraging behaviour in a seabird with reversed sexual dimorphism: the red-footed booby. Oecologia.

[91] Weimerskirch H, Le Corre M, Jaquemet SA, Potier M (2004). Foraging strategy of a top predator in tropical waters: great frigatebirds in the Mozambique Channel. Mar Ecol Prog Ser.

[92] Cairns DK, Bredin KA, Montevecchi WA (1987). Activity budgets and foraging ranges of breeding common murres. Auk.

[93] Dias MP, Granadeiro J, Catry P (2012). Working the day or the night shift? Foraging schedules of Cory’s shearwaters vary according to marine habitat. Mar Ecol Prog Ser.

[94] Drazen JC, De Forest LG, Domokos R (2011). Micronekton abundance and biomass in Hawaiian waters as influenced by seamounts, eddies, and the moon. Deep Sea Res Part I Oceanogr Res Pap.

[95] Boehlert GW, Genin A. A review of the effects of seamounts on biological processes. In Geophysical Monograph. Edited by Keating B, Fryer P, Batiza R, Boehlert GW. American Geophysical Union; 1987:319–344.

[96] Walker WA, Fitzgerald S. Preliminary results on the diet of Laysan albatross and the Use of fisheries by-caught marine birds in investigations of natural feeding strategy. Poster presented at the 40th Annual Pacific Seabird Group Meeting. 2013.

[97] Shaffer SA, Costa DP, Weimerskirch H (2003). Foraging effort in relation to the constraints of reproduction in free‐ranging albatrosses. Funct Ecol.

[98] Phalan B, Phillips RA, Silk JRD, Afanasyev V, Fukuda A, Fox J (2007). Foraging behaviour of four albatross species by night and day. Mar Ecol Ser.

[99] Mackley E, Phillips R, Silk JD, Wakefield E, Afanasyev V, Furness R (2011). At-sea activity patterns of breeding and nonbreeding white-chinned petrels Procellaria aequinoctialis from South Georgia. Mar Biol.

[100] Regular PM, Hedd A, Montevecchi WA (2011). Fishing in the dark: a pursuit-diving seabird modifies foraging behaviour in response to nocturnal light levels. PLoS One.

[101] Amarasekare P, Hoopes MF, Mouquet N, Holyoak M (2004). Mechanisms of coexistence in competitive metacommunities. Am Nat.

[102] Begon M, Townsend CR, Harper JL (2006). Ecology: from individuals to ecosystems.

[103] Wakefield ED, Bodey TW, Bearhop S, Blackburn J, Colhoun K, Davies R (2013). Space partitioning without territoriality in gannets. Science.

[104] Grémillet D, Dell’Omo G, Ryan PG, Peters G, Ropert-Coudert Y, Weeks SJ (2004). Offshore diplomacy, or how seabirds mitigate intra-specific competition: a case study based on GPS tracking of Cape gannets from neighbouring colonies. Mar Ecol Ser.

[105] Sokolowski MB, Pereira HS, Hughes K (1997). Evolution of foraging behavior in Drosophila by density-dependent selection. Proc Natl Acad Sci U S A.

[106] Lewis S, Sherratt TN, Hamer KC, Wanless S (2001). Evidence of intra-specific competition for food in a pelagic seabird. Nature.

[107] Lewis S, Benvenuti S, Dall–Antonia L, Griffiths R, Money L, Sherratt TN, et al. Sex-specific foraging behaviour in a monomorphic seabird. Proc R Soc London B Biol Sci. 2002;269:1687–93. 10.1098/rspb.2002.2083 PMC1691079 12204129

[108] Thaxter CB, Daunt F, Hamer KC, Watanuki Y, Harris MP, Grémillet D, et al. Sex-specific food provisioning in a monomorphic seabird, the common guillemot Uria aalge: nest defence, foraging efficiency or parental effort? J Avian Biol. 2009;40:75–84.

[109] Pinet P, Jaquemet S, Phillips RA, Le Corre M (2012). Sex-specific foraging strategies throughout the breeding season in a tropical, sexually monomorphic small petrel. Anim Behav.

[110] Hedd A, Montevecchi WA, Phillips RA, Fifield DA (2014). Seasonal sexual segregation by monomorphic Sooty Shearwaters Puffinus griseus reflects different reproductive roles during the pre-laying period. PLoS One.

[111] Phillips RA, Silk JRD, Phalan B, Catry P, Croxall JP (2004). Seasonal sexual segregation in two Thalassarche albatross species: competitive exclusion, reproductive role specialization or foraging niche divergence?. Proc Biol Sci.

[112] Stauss C, Bearhop S, Bodey TW, Garthe S, Gunn C, Grecian WJ, et al. Sex-specific foraging behaviour in northern gannets Morus bassanus: incidence and implications. Mar Ecol Prog Ser. 2012;457:151–62.

[113] Rice DW, Kenyon KW (1962). Breeding cycles and behavior of Laysan and Black-footed Albatrosses. Auk.

[114] Arata JA, Sievert P, Naughton M. Status Assessment of Laysan and Black-Footed Albatrosses,North Pacific Ocean, 1923–2005. USGS/USFWS Technical Report. 2009.

[115] Villegas-Amtmann S, Simmons SE, Kuhn CE, Huckstadt LA, Costa DP (2011). Latitudinal range influences the seasonal variation in the foraging behavior of marine top predators. PLoS One.

[116] Thorne LH, Hazen EL, Bograd SJ, Foley DG, Conners MG, Kappes MA, Hyemi K, Tremblay Y, Costa DP, Shaffer SA: Sympatric North Pacific albatross species show contrasting responses to climate variability. Mov Ecol. Accepted September 2015.

[117] Walter ST, Leberg PL, Dindo JJ, Karubian JK (2014). Factors influencing Brown Pelican (Pelecanus occidentalis) foraging movement patterns during the breeding season. J Zool.

[118] Lescroël A, Ballard G, Toniolo V, Barton KJ, Wilson PR, Lyver PO, et al. Working less to gain more: when breeding quality relates to foraging efficiency. Ecology. 2010;91:2044–55. 10.1890/09-0766.1 20715627

[119] Nychka D (1988). Bayesian confidence intervals for smoothing splines. J Am Stat Assoc.

