# Supplementary figures and images for: Shadowed by scale: subtle behavioral niche partitioning in two sympatric, tropical breeding albatross species
Source: Mov Ecol. 2015 Sep 21;3(1):28. doi: 10.1186/s40462-015-0060-7 (PMC4576409; doi:10.1186/s40462-015-0060-7)

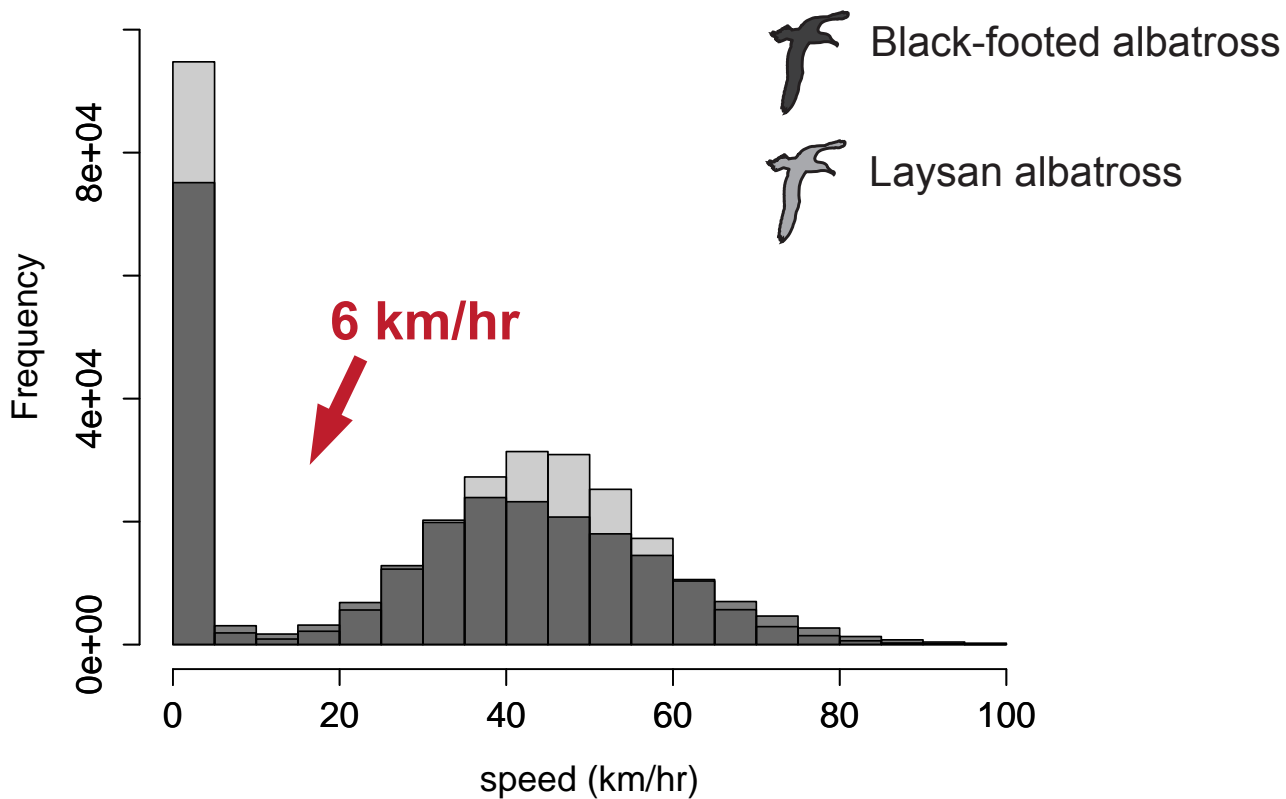

Supplement: Additional file 1: Figure S1. — A bimodal distribution of speeds in both species, indicating a speed threshold of ~6 km/h below which birds do not remain aloft. (PDF 338 kb) [file 40462_2015_60_MOESM1_ESM.pdf]

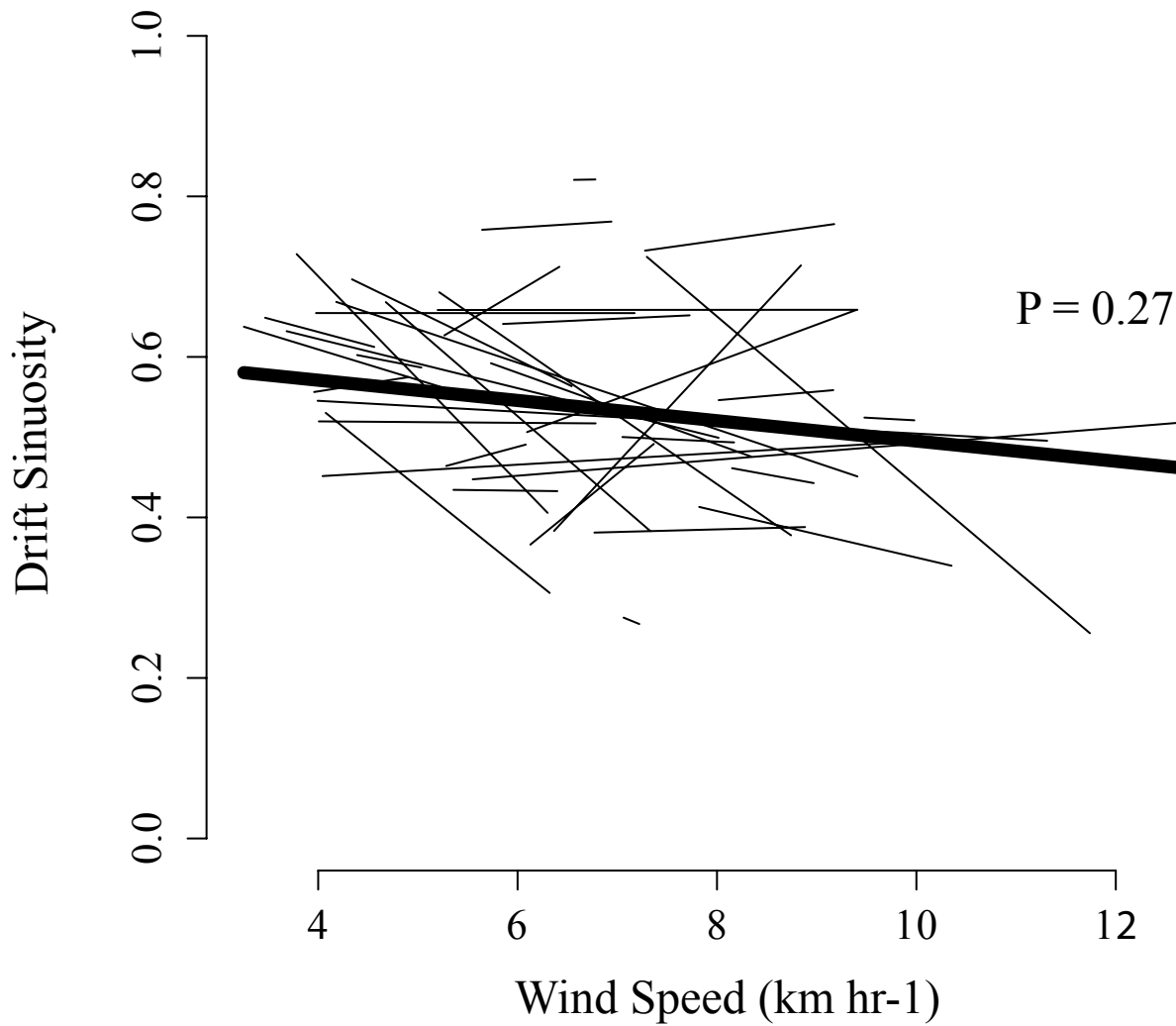

Supplement: Additional file 2: Figure S2. — Wind speed did not affect sinuosity of tracks in drifting birds, validating the use of drift sinuosity as a proxy for drift foraging activity. Wind speeds were extracted at the first location of every drift, so that each drift had an associated wind speed value. A linear mixed model was constructed with drift sinuosity as a continuous response variable to wind speed with species as a fixed factor. Individual bird was included as a random effect since birds had numerous drifts within a foraging trip. Slopes of individual birds were allowed to vary and are represented by the thinner lines, while the population mean slope (of both species) is the bold line. Wind speed, nor species, had a significant effect on sinuosity (t35,655 = −1.11, P = 0.27). (PDF 124 kb) [file 40462_2015_60_MOESM2_ESM.pdf]

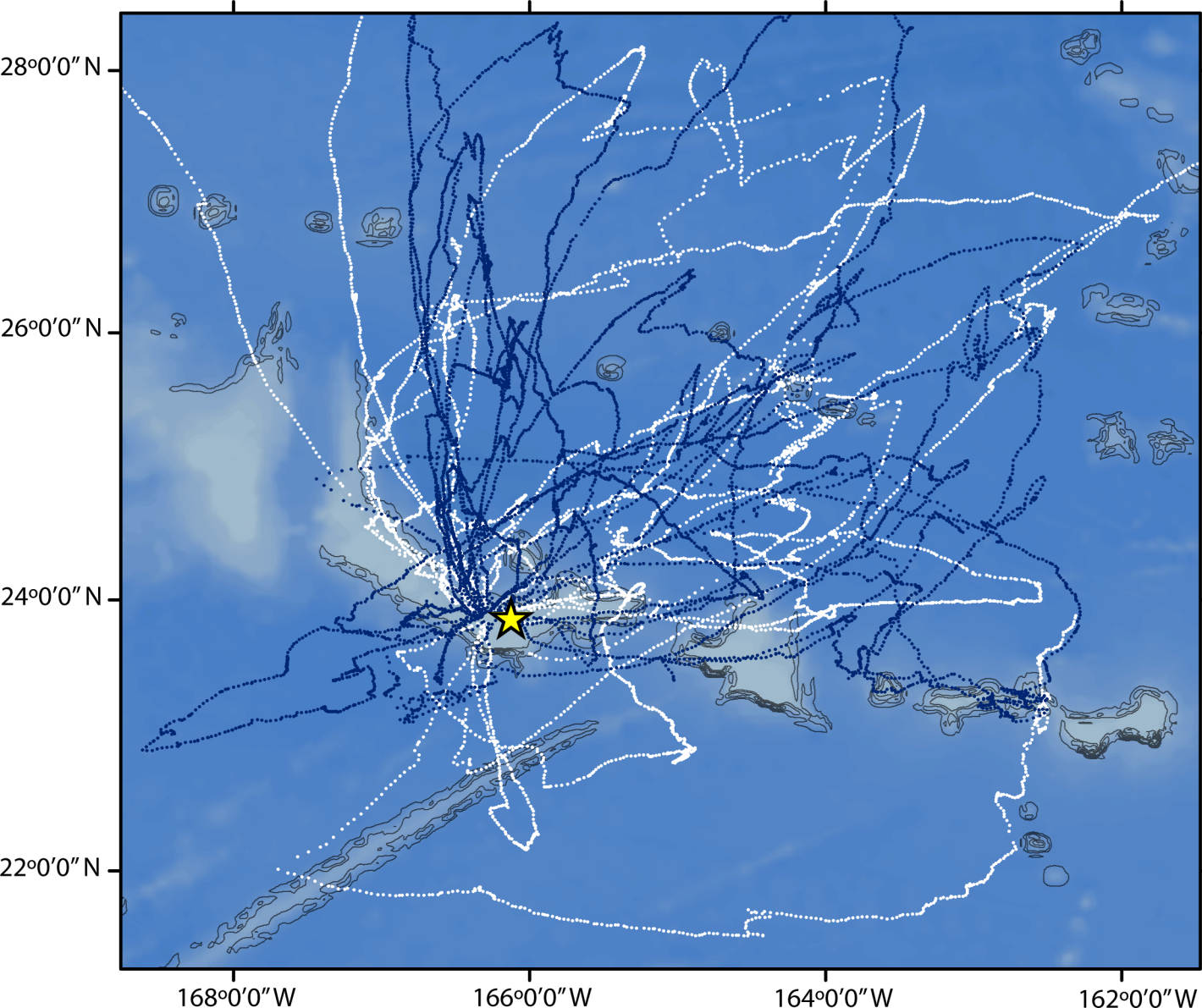

Supplement: Additional file 3: Figure S3. — Black-footed (black) and Laysan (white) albatross GPS tracks deployed at Tern Island (black star) during the brood-guard stage in 2006, 2009, 2010 and 2012. Tracks were subsampled to 1 location every 5 min for the purpose of this illustration. Some individuals of both species visited both the steep bathymetric slope of the northwestern Hawaiian Islands as well as deeper submerged seamounts in pelagic waters. Both species predominately foraged north and northeast of Tern Island with a few individuals from both species foraging south and southwest. The vast majority of trip durations were spent in pelagic waters. (PDF 5217 kb) [file 40462_2015_60_MOESM3_ESM.pdf]
