# Supplementary material for: Shadowed by scale: subtle behavioral niche partitioning in two sympatric, tropical breeding albatross species
Source: Mov Ecol. 2015 Sep 21;3(1):28. doi: 10.1186/s40462-015-0060-7 (PMC4576409; doi:10.1186/s40462-015-0060-7)
Supplement: Additional file 4: Table S1. — Mass and morphometrics (means ± SD) of Laysan and black-footed albatross breeding on Tern Island. (DOCX 13 kb) [file 40462_2015_60_MOESM4_ESM.docx]

**Supplemental Table 1.** Mass and morphometrics (means ± SD) of Laysan and black-footed albatross breeding on Tern Island.

|  | Laysan albatross (163) | | Black-footed albatross (167) | |
| --- | --- | --- | --- | --- |
|  | Female (77) | Male (86) | Female (80) | Male (87) |
| Mass Initial (kg) | 2.37 ± .03 | 2.52 ± .03 | 2.76 ± .04 | 3.01 ± .04 |
| Tarsus (cm) | 10.32 ± .04 | 10.61 ± .05 | 10.81 ± .05 | 11.48 ± .04 |
| Culmen (cm) | 10.64 ± .03 | 11.37 ± .04 | 10.23 ± .05 | 10.78 ± .04 |
| Minimum Bill Depth (cm) | 2.25 ± .01 | 2.38 ± .01 | 2.53 ± .01 | 2.68 ± .01 |
| Maximum Bill Depth (cm) | 2.40 ± .01 | 2.57 ± .01 | 2.68 ± .01 | 2.87 ± .01 |
